# Tomato root-associated *Sphingobium* harbors genes for catabolizing toxic steroidal glycoalkaloids

**DOI:** 10.1128/mbio.00599-23

**Published:** 2023-09-29

**Authors:** Masaru Nakayasu, Kyoko Takamatsu, Keiko Kanai, Sachiko Masuda, Shinichi Yamazaki, Yuichi Aoki, Arisa Shibata, Wataru Suda, Ken Shirasu, Kazufumi Yazaki, Akifumi Sugiyama

**Affiliations:** 1 Research Institute for Sustainable Humanosphere, Kyoto University, Uji, Kyoto, Japan; 2 Plant Immunity Research Group, RIKEN Center for Sustainable Resource Science, Yokohama, Kanagawa, Japan; 3 Tohoku Medical Megabank Organization, Tohoku University, Sendai, Miyagi, Japan; 4 Graduate School of Information Sciences, Tohoku University, Sendai, Miyagi, Japan; 5 Laboratory for Microbiome Sciences, RIKEN Center for Integrative Medical Sciences, Yokohama, Kanagawa, Japan; California Institute of Technology, Pasadena, California, USA; Stanford University, Stanford, California, USA

**Keywords:** degradation enzymes, rhizosphere, steroid-type saponins, *Sphingobium*, tomato

## Abstract

Plant roots exude various organic compounds, including plant specialized metabolites (PSMs), into the rhizosphere. The secreted PSMs enrich specific microbial taxa to shape the rhizosphere microbiome, which is crucial for the healthy growth of the host plants. PSMs often exhibit biological activities; in turn, some microorganisms possess the capability to either resist or detoxify them. Saponins are structurally diverse triterpene-type PSMs that are mainly produced by angiosperms. They are generally considered as plant defense compounds. We have revealed that α-tomatine, a steroid-type saponin secreted from tomato (*Solanum lycopersicum*) roots, increases the abundance of *Sphingobium* bacteria. To elucidate the mechanisms underlying the α-tomatine-mediated enrichment of *Sphingobium*, we isolated *Sphingobium* spp. from tomato roots and characterized their saponin-catabolizing abilities. We obtained the whole-genome sequence of *Sphingobium* sp. RC1, which degrades steroid-type saponins but not oleanane-type ones, and performed a gene cluster analysis together with a transcriptome analysis of α-tomatine degradation. The *in vitro* characterization of candidate genes identified six enzymes that hydrolyzed the different sugar moieties of steroid-type saponins at different positions. In addition, the enzymes involved in the early steps of the degradation of sapogenins (i.e., aglycones of saponins) were identified, suggesting that orthologs of the known bacterial steroid catabolic enzymes can metabolize sapogenins. Furthermore, a comparative genomic analysis revealed that the saponin-degrading enzymes were present exclusively in certain strains of *Sphingobium* spp., most of which were isolated from tomato roots or α-tomatine-treated soil. Taken together, these results suggest a catabolic pathway for highly bioactive steroid-type saponins in the rhizosphere.

**IMPORTANCE:**

Saponins are a group of plant specialized metabolites with various bioactive properties, both for human health and soil microorganisms. Our previous works demonstrated that *Sphingobium* is enriched in both soils treated with a steroid-type saponin, such as tomatine, and in the tomato rhizosphere. Despite the importance of saponins in plant–microbe interactions in the rhizosphere, the genes involved in the catabolism of saponins and their aglycones (sapogenins) remain largely unknown. Here we identified several enzymes that catalyzed the degradation of steroid-type saponins in a *Sphingobium* isolate from tomato roots, RC1. A comparative genomic analysis of *Sphingobium* revealed the limited distribution of genes for saponin degradation in our saponin-degrading isolates and several other isolates, suggesting the possible involvement of the saponin degradation pathway in the root colonization of *Sphingobium* spp. The genes that participate in the catabolism of sapogenins could be applied to the development of new industrially valuable sapogenin molecules.

## INTRODUCTION

Plant roots secret an array of organic compounds, including biologically active plant specialized metabolites (PSMs), into the rhizosphere, which is the zone of soil surrounding the roots (1). The secreted PSMs have a wide range of ecological functions that allow them to mediate the interaction between the host plants and their surrounding organisms (2, 3). In the last decade, PSMs have been reported to shape microbial communities called the microbiome, and to enrich specific taxa in the rhizosphere and roots (4 – 6). Moreover, the root-associated microbiome formed by PSMs can improve the growth of the host plants under environmental stresses, such as nutrient deficiency, dryness, and attack by pathogens and herbivores (7 – 11).

**Fig 1:**
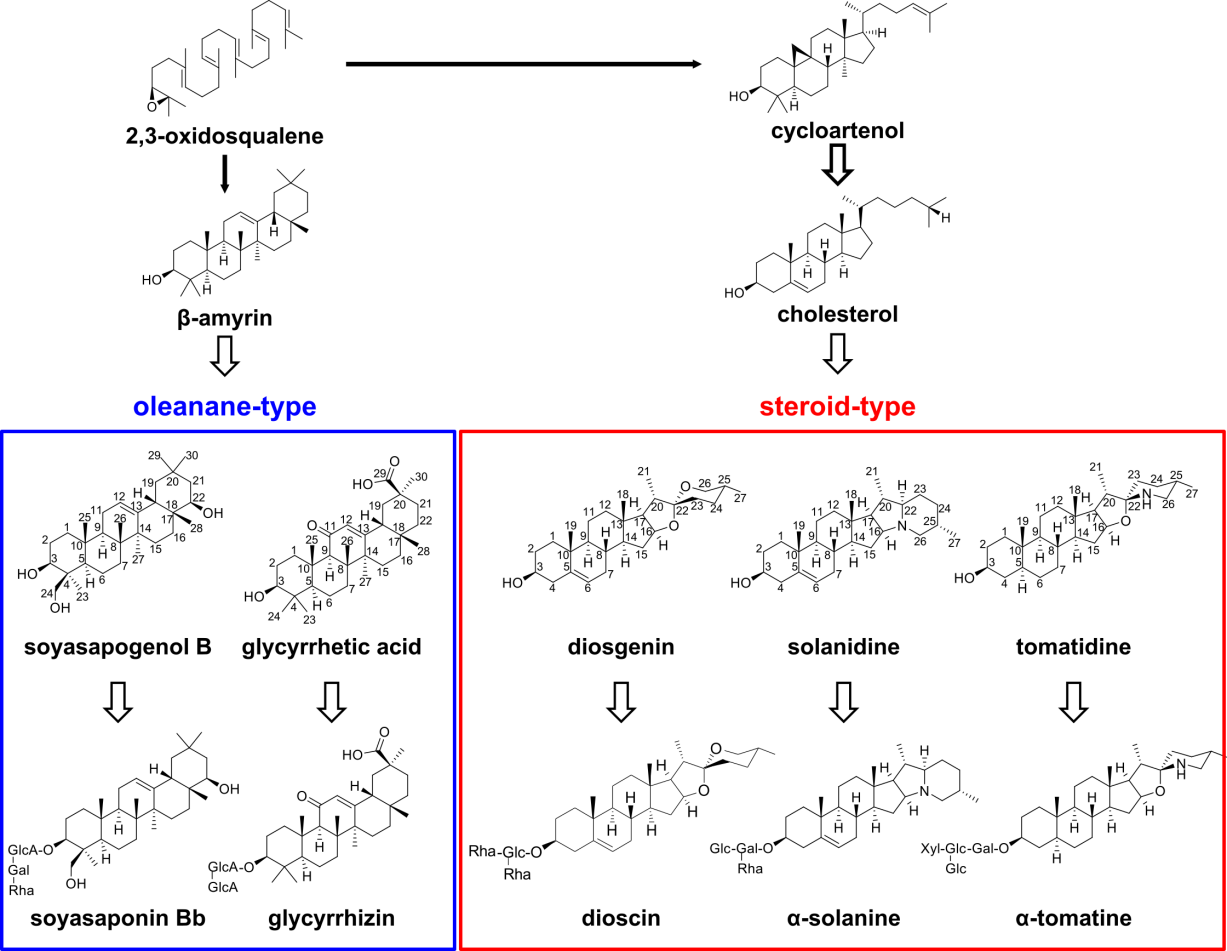
Biosynthesis of steroid- and oleanane-type saponins in plants. The solid and white arrows indicate one and multiple reaction steps, respectively.

Saponins are PSMs with diverse chemical structures (Fig. 1) that act as natural surfactants and are broadly distributed in angiosperm plants (12). The biosynthesis of saponins is derived from the mevalonate pathway and is initiated by the formation of a triterpenoid backbone, followed by further chemical decorations, such as oxidation and glycosylation (13, 14). Structural diversity is provided by the variable cyclization pattern of 2,3-oxidosqualene, as the last common precursor of all triterpenes except hopanoids, which are directly formed from squalene; furthermore, the diverse cyclizations of saponins are catalyzed by the oxidosqualene cyclase (OSC) group of enzymes. For example, the cucurbitadienol (cucurbitane-type), dammarenediol-II (dammarane-type), and β-amyrin (oleanane-type) compounds are formed by specific OSCs and are finally converted to ginsenosides, mogrosides, and soyasaponins, which are triterpenoid saponins of Chinese ginseng (*Panax notoginseng*), monk fruit (*Siraitia grosvenorii*), and several *Fabaceae* plants (including soybean [*Glycine max*]), respectively (15). Glycyrrhizin is another oleanane-type triterpenoid saponin that is used as a natural sweetener and is found only in licorice (*Glycyrrhiza* spp., *Fabaceae*) (16). In addition, the cyclization to cycloartenol eventually leads to the production of steroid-type saponins via cholesterol synthesis; these saponins are classified into two groups: steroidal saponins and steroidal glycoalkaloids (SGAs), with the former including dioscin in *Dioscorea* plants (17) and the latter encompassing a nitrogen atom in molecules that mainly occur as α-tomatine in *Solanum lycopersicum* (tomato) and as α-solanine and α-chaconine in *S. tuberosum* (potato) (18, 19).

According to the molecular species, saponins exhibit foaming and emulsifying properties; hemolytic and cytotoxic activities; pharmacological actions; taste properties; and toxicity against microbes, insects, and mollusks (20). Moreover, because of their allelopathic activities and inhibitory effects against plant pathogens and herbivores, saponins are thought to serve as chemical-defense compounds that protect the host plants (21 – 23). For instance, a tomato mutant with a low α-tomatine content exhibited increased susceptibility to the larvae of the generalist herbivore *Spodoptera litura* (24). In oat (*Avena strigosa*), a mutant lacking avenacin, which is an oleanane-type triterpenoid, showed impaired resistance to various fungal pathogens (25). Our previous works have shown that soybean (*Glycine max*) and tomato secrete soyasaponins and α-tomatine from their roots into the rhizosphere, and that soyasaponins and α-tomatine specifically enriched the rhizosphere in *Novosphingobium* and *Sphingobium*, respectively, both of which are genera belonging to family *Sphingomonadaceae* (26 – 28). These studies revealed new functions of saponin in microbiome formation, in addition to their roles in host plant defense, as well as a relationship between their chemical structures and their biological effects (29).

Saponins secreted into the rhizosphere are degraded by soil microorganisms. *Arthrobacter* and *Serratia* isolated from the soils surrounding green potato peel degraded α-solanine and α-chaconine (30), whereas *Sphingobium* spp. isolated from tomatine-treated soil degraded α-tomatine (28). The aglycones of saponins, such as solanidine and tomatidine (which are collectively called sapogenins), do not accumulate in these bacteria, suggesting that soil bacteria have the ability to metabolize sapogenins. In contrast, intestinal microorganisms can hydrolyze the glycoside bonds of saponins to produce sapogenins, which are not further degraded; rather, they are adsorbed from the intestine (31 – 33). These findings suggest that soil bacteria, in contrast with intestinal bacteria, possess unique catabolic enzymes for the degradation of saponins. Many microbial glycoside hydrolases (GHs), which catalyze saponin deglycosylation steps, have been identified, especially from fungal pathogens (34 – 38). In contrast, the microbial metabolic pathway for sapogenin degradation has not been uncovered. The steroid (but not sapogenin)-degradation enzymes that are present in *Comamonas testosteroni* TA441 have been well studied; moreover, their orthologs in the *Novosphingobium tardaugens* strain ARI-1 (NBRC 16725) have also been identified via *in silico* analysis (39, 40). In the present study, we sequenced the genome of *Sphingobium* spp. isolated from tomato roots as well as α-tomatine-treated soils, and identified the enzymes responsible for the degradation of both the sugar moieties and the tomatidine backbone of α-tomatine. A comparative genomic analysis revealed the limited distribution of α-tomatine-metabolizing genes in *Sphingobium*, suggesting the molecular mechanism via which root-associated microbes manage the bioactive PSMs secreted from host plants.

## RESULTS

### Isolation of *Sphingobium* spp. and their saponin degradation activities

**Fig 2:**
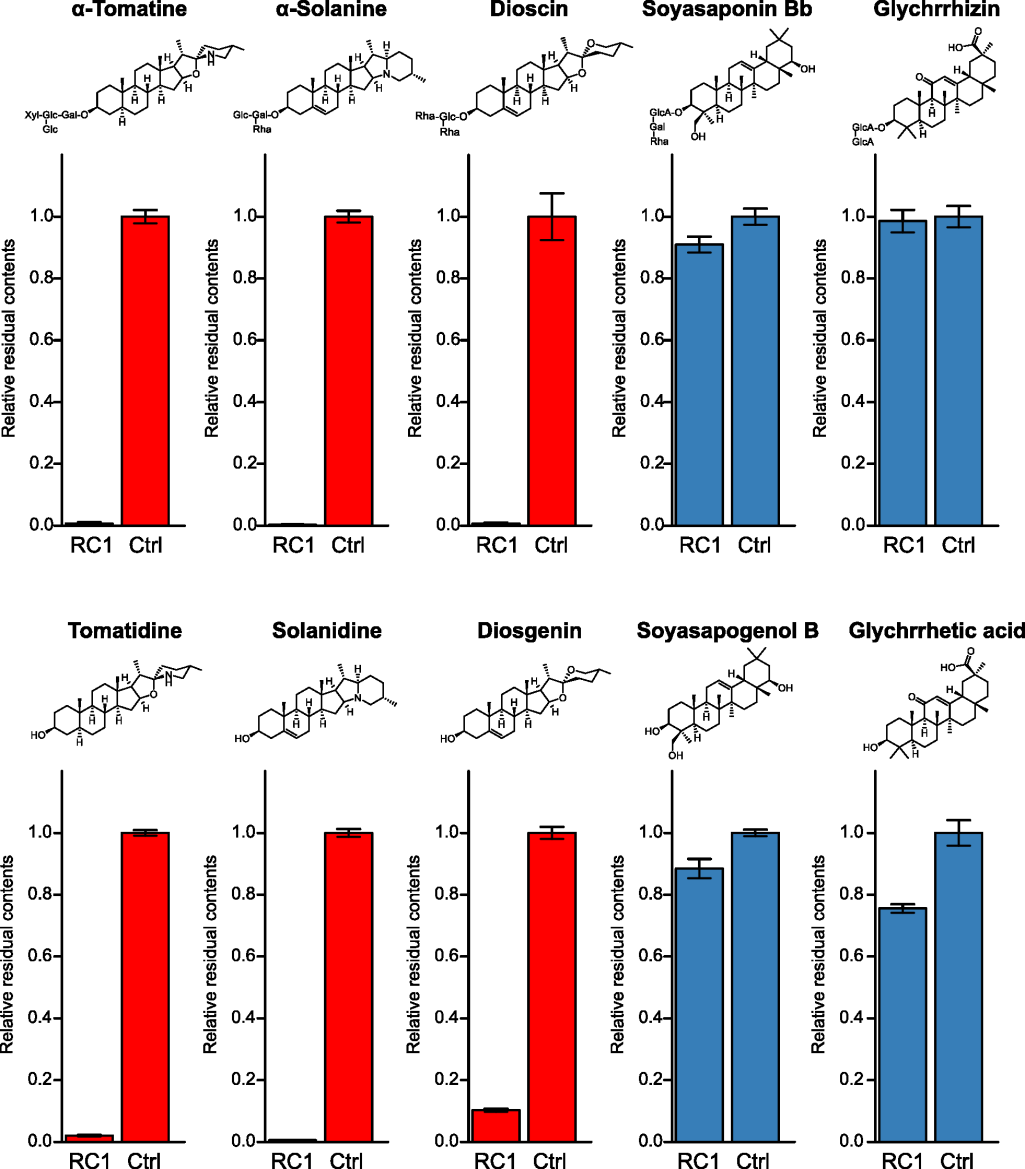
Substrate specificity of the saponin- and sapogenin-degradation activities in RC1 resting cells. The residual substrate contents in the reaction mixture incubated for 3 h are shown relative to that obtained without RC1, as the negative control (Ctrl). The error bars indicate the standard deviation (*n* = 3, technical replicates).

We reported previously that three *Sphingobium* strains isolated from α-tomatine-treated soils degraded α-tomatine (28). Here, we isolated additional 11 *Sphingobium* strains from α-tomatine-treated soils and one strain from tomato roots (Table S1). We then measured the α-tomatine-degradation activities using those *Sphingobium* strains. The incubation of these resting cells with α-tomatine (as a substrate) revealed that all strains degraded this compound (Fig. S1). Using one strain from tomato roots termed RC1, we evaluated the substrate specificity of its degradation activities toward several saponins and their aglycones (Fig. 1). RC1 degraded steroid-type saponins, i.e., α-tomatine, α-solanine, and dioscin; as well as their respective sapogenins: tomatidine, solanidine, and diosgenin (Fig. 2). In turn, RC1 did not degrade oleanane-type saponins, i.e., soyasaponin Bb and glycyrrhizin; or the former sapogenin: soyasapogenol B (Fig. 2). Next, we assessed the time course of α-tomatine degradation by RC1 (Fig. S2). With a reaction time of 90 min, RC1 degraded α-tomatine into several products, including two peaks with retention times of 6.6 and 5.2 min, respectively. The former gave a major mass fragment ion at *m/z* 416, identical to authentic tomatidine; whereas the latter exhibited a parental ion at *m/z* 740 that was estimated to be γ-tomatine, in which one molecule each of d-glucose and d-xylose were removed from α-tomatine. RC1 completely degraded α-tomatine and its degradation intermediates within 180 min. Therefore, it was predicted that *Sphingobium* first hydrolyzes the oligosaccharide parts of steroid-type saponins in a stepwise manner, followed by sapogenin degradation.

### Selection of candidate genes responsible for saponin degradation via whole-genome analysis

We performed whole-genome sequencing of RC1 and 14 isolates from α-tomatine-treated soils, to identify the genes involved in the degradation of steroid-type saponins in *Sphingobium*. The 16S rRNA V4 regions of all strains but TomTYG65, TomTYG72, TomTYG74, and TomMM15 were identical to those of the amplicon sequence variant (ASV) belonging to *Sphingobium*, which were remarkably increased by steroid-type saponins (28, 41) (Fig. S3). The total sequence length of all isolates ranged from 3.5 to 4.4 Mbp, which was comparable to that of closely related strains (Data Set S1). BUSCO assessments (42) classified all obtained genome assemblies as being of high quality (completeness score >99%) (Data Set S1). We searched for genes encoding steroid-type saponin degradation enzymes in the RC1 genome, from which 3,627 coding sequences (CDSs) were annotated using the Prokka pipeline (43) (Data Set S1).

#### GHs for saponin degradation

A total of 33 glycoside hydrolases (GHs) were extracted from the RC1 genome and were classified into specific GH families using dbCAN2, which is a meta server for automated carbohydrate-active enzyme (CAZyme) annotation (44) (Data Set S2). In addition, we found that a total of six GH genes existed in close proximity in two regions of the RC1 genome and belonged to GH families 3, 39, 78, and 106 (Fig. S4). Glycoside hydrolases that cleave the glycoside bonds in ginsenosides, called ginsenosidases, have been identified from several microorganisms and belong to GH families 1, 2, 3, 10, 39, 42, 51, and 78 (45). The GH106 family from *Novosphingobium* sp. PP1Y reportedly exhibits rhamnosidase activity toward flavonoid glycosides (46). Therefore, we selected NNNEINPD_01945, NNNEINPD_01944, NNNEINPD_01942, NNNEINPD_03247, NNNEINPD_03248, and NNNEINPD_03250 as steroid-type saponin GH candidates, and termed them *SpGH3-4*, *SpGH39-1*, *SpGH3-3*, *SpGH3-1*, *SpGH106-1*, and *SpGH78-1*, respectively (Fig. S4).

Previously, tomatinases produced by tomato pathogens, such as *Fusarium oxysporum* f. sp. *lycopersici*, were shown to be extracellular enzymes that hydrolyze α-tomatine by cleaving the glycoside bond (35). Our *in silico* analysis using SignalP 6.0, version 0.0.52 (47), strongly suggested that the six GH candidates contain signal peptides at their N termini. SpGH3-1, SpGH78-1, and SpGH106-1 were predicted to have signal peptides for the general secretion protein export pathway, called Sec/SPI, whereas SpGH3-3, SpGH3-4, and SpGH39-1 were predicted to have signal peptides for the twin-arginine translocation pathway, called Tat/SPI.

#### Enzymes for sapogenin degradation

We hypothesized that those orthologs of steroid degradation enzymes in RC1 are involved in the degradation of steroid-type sapogenins. Functional annotation using Kofam KOALA assigned several proteins of RC1 that were involved in the conversion of 5α-androstane-3,17-dione and androst-5-en-3,17-dione to 4,5:9,10-diseco-3-hydroxy-5,9,17-trioxoandrosta-1(10),2-diene-4-oate in the KEGG pathway map for steroid degradation (Fig. S5). This result indicated that RC1 carries a set of steroid degradation genes, which were predicted here to also participate in sapogenin degradation.

C3 oxidation is considered to be the initial reaction step in the microbial degradation of steroids with a hydroxyl group at the C-3 position. However, no RC1 proteins were assigned to this reaction in the KEGG pathway (Fig. S5). To identify the enzymes that are responsible for the initial reaction step of sapogenin degradation, we performed a BLAST search against all protein sequences in RC1 using the 3,17β-hydroxysteroid dehydrogenase (3,17β-HSD) encoded by the EGO55_02230 gene in *N. tardaugens* ARI-1 as a query (40). NNNEINPD_03057 and NNNEINPD_02694 in RC1 exhibited 71% and 54% amino acid identities with EGO55_02230, respectively, and shared 55% amino acid identity with each other. We selected NNNEINPD_03057 and NNNEINPD_02694 as candidates for sapogenin 3β-hydroxysteroid dehydrogenase (3βHSD), and designated them Sp3βHSD1 and Sp3βHSD2, respectively.

In steroid metabolism, the 3βHSDs in a wide range of organisms are often bifunctional enzymes that oxidize Δ5-3-hydroxysteroids at C3, to form Δ5-3-ketosteroids, then isomerize them to form Δ4-3-ketosteroids. In turn, Δ4-3-ketosteroids are also formed by 3-ketosteroid-Δ4-dehydrogenase (3KSΔ4DH), which catalyzes the Δ4-dehydrogenation of 3-ketosteroids containing a single bond between C5 and C6. A BLAST search showed that the 3KSΔ4DH encoded by EGO55_13615 in *N. tardaugens* ARI-1 (40) exhibited an amino acid identity of 46% with NNNEINPD_01949 from RC1. The four 3KSΔ4DH orthologs were assigned to the corresponding reaction in the KEGG pathway (Fig. S5). NNEINPD_01949 is located in the proximity of SpGH3-4, SpGH39-1, and SpGH3-3 in the RC1 genome. Therefore, we selected NNNEINPD_01949 as the candidate enzyme for sapogenin degradation, and termed it Sp3KSΔ4DH1.

### Comparative transcriptome analysis of α-tomatine-treated and mock-treated RC1

In several tomato pathogens, tomatinase activity and the expression of tomatinase genes have been reported to be induced by α-tomatine treatment (37, 48, 49). We investigated whether an α-tomatine-degrading activity was similarly induced in RC1. Treatment with α-tomatine enhanced the degradation activity of RC1 cells (Fig. S6), suggesting that RC1 induces the expression of the degradation enzymes in response to saponin secretion by plants. A comparative transcriptome analysis between α-tomatine-treated and mock-treated RC1 revealed that the transcript levels of the genes encoding the six saponin GH candidates, Sp3βHSD1, Sp3βHSD2, and six ortholog groups assigned to the steroid degradation pathway were expressed at higher levels in α-tomatine-treated vs. mock-treated RC1 (Table S2). The increased expression of these genes in α-tomatine-treated cells indicated that they were potential candidates for further characterization.

### *In vitro* functional analysis of steroid-type saponin GH candidates

**Fig 3:**
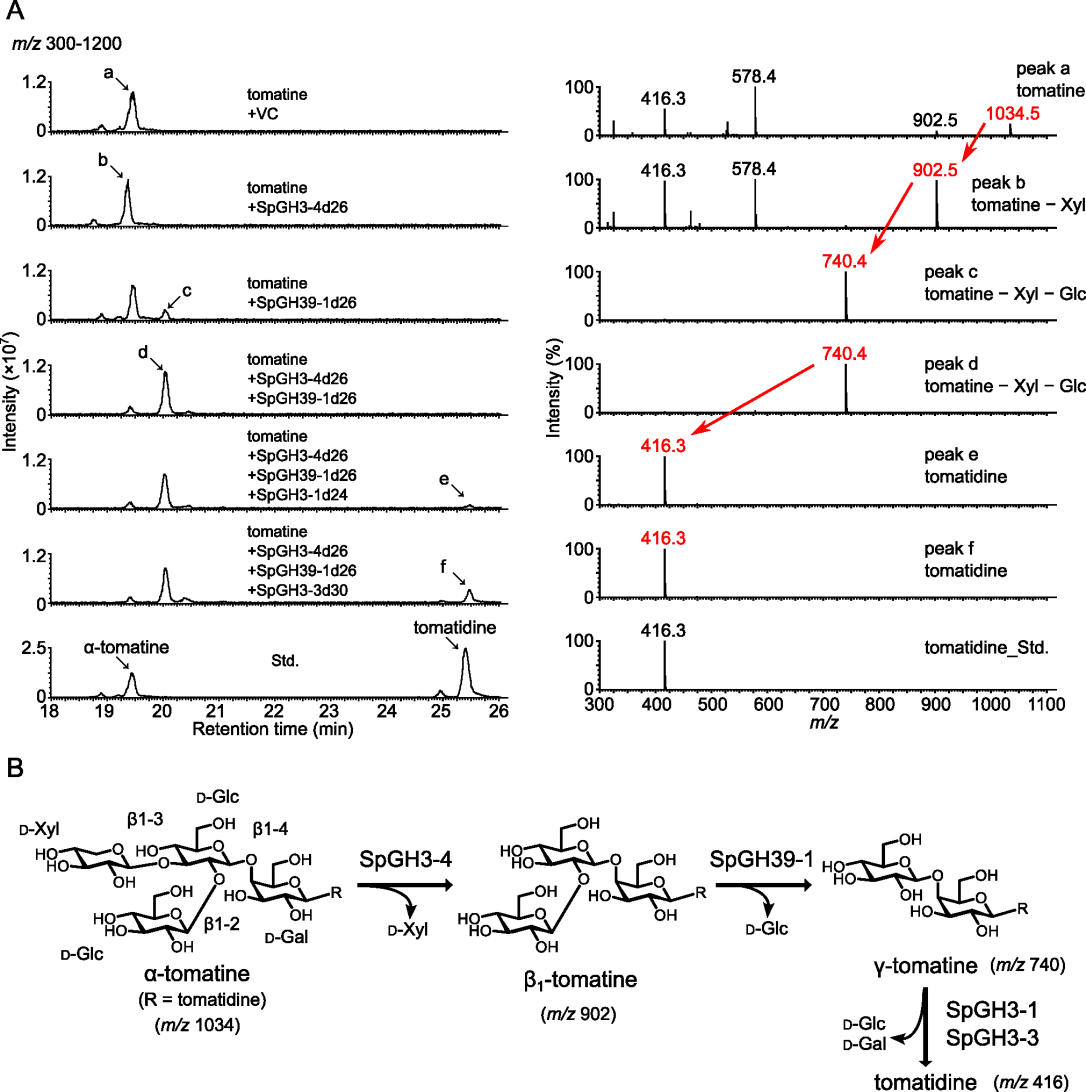
Enzymatic activities of SpGH3-1, SpGH3-3, SpGH3-4, and SpGH39-1 toward α-tomatine. (**A**) LC-MS analysis of the reaction products obtained from the recombinant proteins of SpGH3-1, SpGH3-3, SpGH3-4, and SpGH39-1 using α-tomatine as a substrate. A purified protein from *Escherichia coli* transformed with an empty pET22b vector was used as the negative control (VC). Representative data of the enzymatic activities measured in biological duplicates are shown. The total ion current chromatogram obtained in the positive ionization mode with a full-scan range of *m/z* 300–1200 is shown. The mass spectra of peak a (substrate, α-tomatine) and peaks b–f (reaction products), and tomatidine standard, indicated by arrows in the chromatogram, are shown. The red letters represent the parental ion mass given by the reaction products. (**B**) Proposed enzymatic conversion of α-tomatine to tomatidine, as predicted by the mass spectra of the reaction products.

To investigate the catalytic activities of steroid-type saponin GH candidates, the predicted mature forms of the candidates, in which the N-terminal predicted signal peptides were truncated, were expressed in *Escherichia coli*. The enzymatic activities of the candidates were examined using various saponins as substrates (Fig. 3; Fig. S7 and S8; Table S3).

The use of α-tomatine as a substrate led to its conversion by SpGH3-4 to a product with a retention time of 19.4 min and a mass fragment ion at *m/z* 902.5, which was 132 mass smaller than that of α-tomatine, suggestive of β_1_-tomatine (Fig. 3A). Although incubation with SpGH39-1 left most of the α-tomatine in the assay mixture, a reaction product detected at a retention time of 20.0 min gave a mass fragment ion at *m/z* 740.4, which was 294 mass smaller than that of α-tomatine, suggestive of γ-tomatine (Fig. 3A). Moreover, coincubation with SpGH3-4 and SpGH39-1 metabolized most of α-tomatine to γ-tomatine (Fig. 3A). These results indicate that SpGH3-4 hydrolyzed the β1–3 linked d-xylose from α-tomatine, to produce β_1_-tomatine, and that SpGH39-1 mainly hydrolyzed the β1–2 linked d-glucose from β_1_-tomatine, to produce γ-tomatine (Fig. 3B). Furthermore, coincubation of SpGH3-1 with SpGH3-4 and SpGH39-1 gave a peak with a retention time of 25.5 min and a mass fragment ion at *m/z* 416.3, which was found to be identical to that of tomatidine (Fig. 3A). Finally, coincubation of SpGH3-3 with SpGH3-4 and SpGH39-1 also yielded tomatidine (Fig. 3A). These results suggest that SpGH3-1 and SpGH3-3 hydrolyzed the β-d-glucosyl-(1→4)-β-d-galactosyl moiety from γ-tomatine to produce tomatidine (Fig. 3B).

We next measured the enzymatic activities of GH candidates using other saponins as substrates. In brief, SpGH106-1 hydrolyzed α1-2 linked l-rhamnose from α-solanine (Fig. S7B), whereas SpGH3-1 and SpGH3-3 hydrolyzed β1-3 linked d-glucose from β_2_-solanine and then d-galactose from γ-solanine, to eventually produce solanidine (Fig. S7B). SpGH106-1 and SpGH78-1 probably hydrolyzed α1-2- and α1-4-linked l-rhamnose from dioscin, respectively, and SpGH3-1 subsequently hydrolyzed d-glucose from diosgenin-3-*O*-β-d-glucoside, to produce diosgenin (Fig. S8B).

The use of soyasaponin Bb and glycyrrhizin as substrates did not yield any product peaks from the six saponin GH candidates, indicating that they are specific GHs for steroid-type saponins (Table S3). Moreover, SpGH3-4 and SpGH39-1 did not recognize α-solanine and dioscin as substrates, and SpGH78-1 and SpGH106-1 did not metabolize α-tomatine (Table S3).

### *In vitro* functional analysis of Sp3βHSD1, Sp3βHSD2, and Sp3KSΔ4DH1 toward pregnane derivatives and sapogenins

Recombinant proteins of Sp3βHSD1, Sp3βHSD2, and Sp3KSΔ4DH1 were prepared using a bacterial expression system in *E. coli*, for use in *in vitro* assays. First, their enzymatic activities were analyzed using pregnane derivatives as substrates (Fig. S9 to S11). Sp3βHSD1 and Sp3βHSD2 converted isopregnanolone to a product with a retention time of 11.2 min and a major mass fragment ion at *m/z* 317.3, which was identical to that of 5α-pregnane-3,20-dione (Fig. S9). Subsequently, they also converted pregnenolone to a product with a retention time of 10.2 min and a major mass fragment ion at *m/z* 315.3, which was identical to that of progesterone (Fig. S10). Sp3KSΔ4DH1 metabolized 5α-pregnane-3,20-dione to a product with a retention time of 10.2 min and a major mass fragment ion at *m/z* 315.3, which was identical to that of progesterone (Fig. S11). These results indicated that Sp3βHSD1 and Sp3βHSD2 catalyzed the C3 oxidation and Δ5-Δ4 isomerization of pregnane derivatives, and that Sp3KSΔ4DH1 catalyzed their Δ4-dehydrogenation.

**Fig 4:**
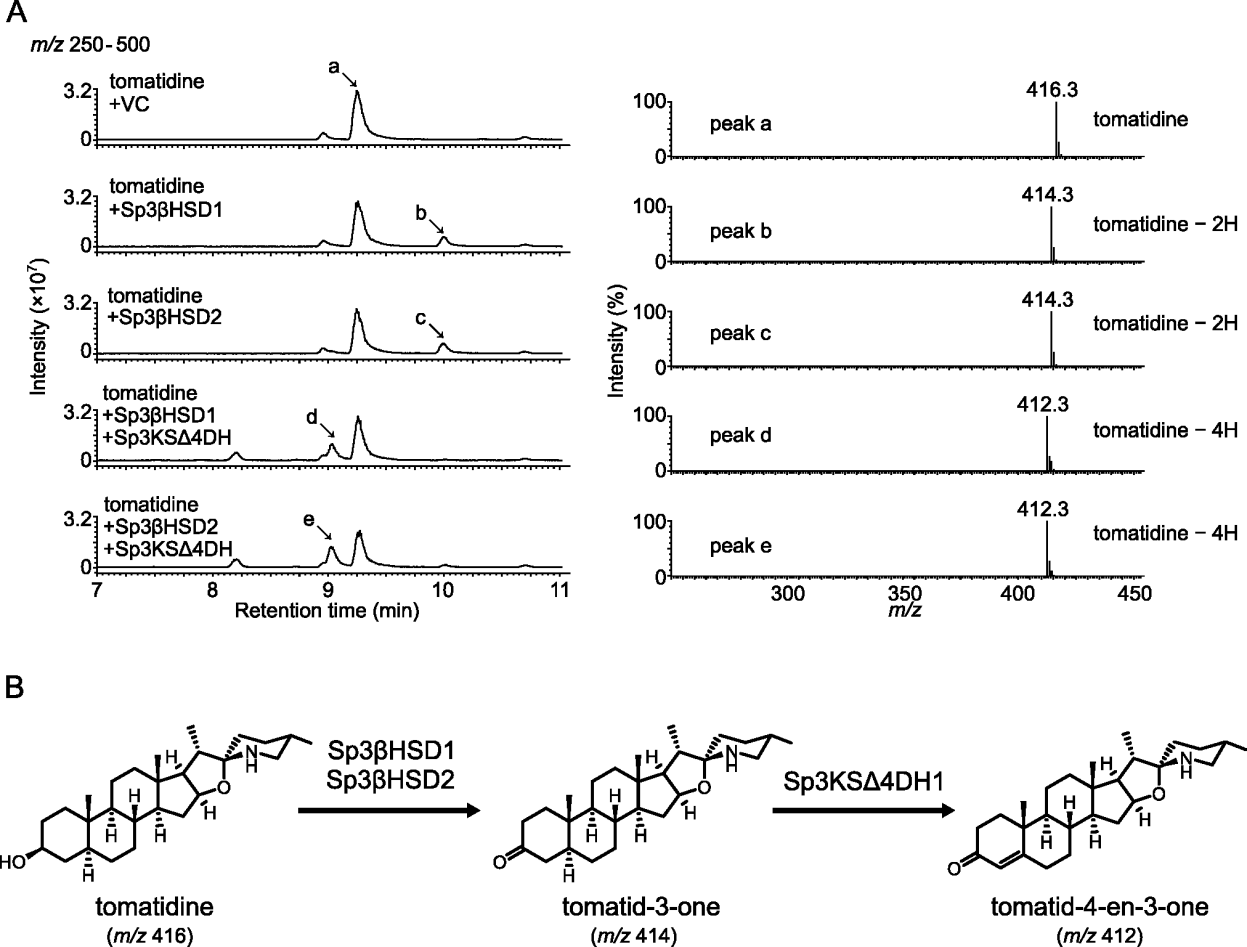
Enzymatic activities of Sp3βHSD1, Sp3βHSD2, and Sp3KSΔ4DH1 toward tomatidine. (**A**) LC-MS analysis of the reaction products obtained from the recombinant proteins of Sp3βHSD1, Sp3βHSD2, and Sp3KSΔ4DH1 using tomatidine as a substrate. A purified protein from *Escherichia coli* transformed with an empty pET22b vector was used as the negative control (VC). Representative data of the enzymatic activities measured in biological duplicates are shown. The total ion current chromatogram obtained in the positive ionization mode with a full-scan range of *m/z* 250–500 is shown. The mass spectra of peak a (substrate, tomatidine) and peaks b–e (reaction products), as indicated by arrows in the chromatogram, are shown. (**B**) Proposed enzymatic conversion of tomatidine to tomatid-4-en-3-one, as predicted by the enzymatic activities of Sp3βHSD1 and Sp3βHSD2 toward isopregnanolone (Fig. **S9**) and of Sp3KSΔ4DH1 toward 5α-pregnane-3,20-dione (Fig. **S11**).

Next, the enzymatic activities of Sp3βHSD1, Sp3βHSD2, and Sp3KSΔ4DH1 toward several sapogenins were surveyed (Fig. 4; Fig. S12 to S15). The use of tomatidine as a substrate led to a reaction product from Sp3βHSD1 and Sp3βHSD2 with a retention time of 10.0 min and a major mass fragment ion at *m/z* 414.3, which was two mass smaller than that of tomatidine (used as the substrate) (Fig. 4A). In turn, coincubation of Sp3KSΔ4DH1 with either Sp3βHSD1 or Sp3βHSD2 produced a peak with a retention time of 9.0 min and a major mass fragment ion at *m/z* 412.3, which was four mass smaller than that of tomatidine (Fig. 4A). Based on their enzymatic activities toward pregnane derivatives, it was suggested that Sp3βHSD1 and Sp3βHSD2 converted tomatidine to tomatid-3-one, which was then metabolized to tomatid-4-en-3-one by Sp3KSΔ4DH1 (Fig. 4B). Similarly, using solanidine, diosgenin, and glycyrrhetic acid as substrates, Sp3βHSD1 and Sp3βHSD2 produced peaks with major mass fragment ions that were two mass smaller than their respective substrates (Fig. S12 to S14). These results suggest that Sp3βHSD1 and Sp3βHSD2 converted solanidine, diosgenin, and glycyrrhetic acid to solanid-4-en-3-one, diosgen-4-en-3-one, and 3-keto-glycyrrhetic acid, respectively. In contrast, soyasapogenol B was not recognized as a substrate for Sp3βHSD1 and Sp3βHSD2 (Fig. S15).

### Distribution of the genes encoding steroid-catabolizing enzymes and saponin GHs in *Sphingobium* spp.

**Fig 5:**
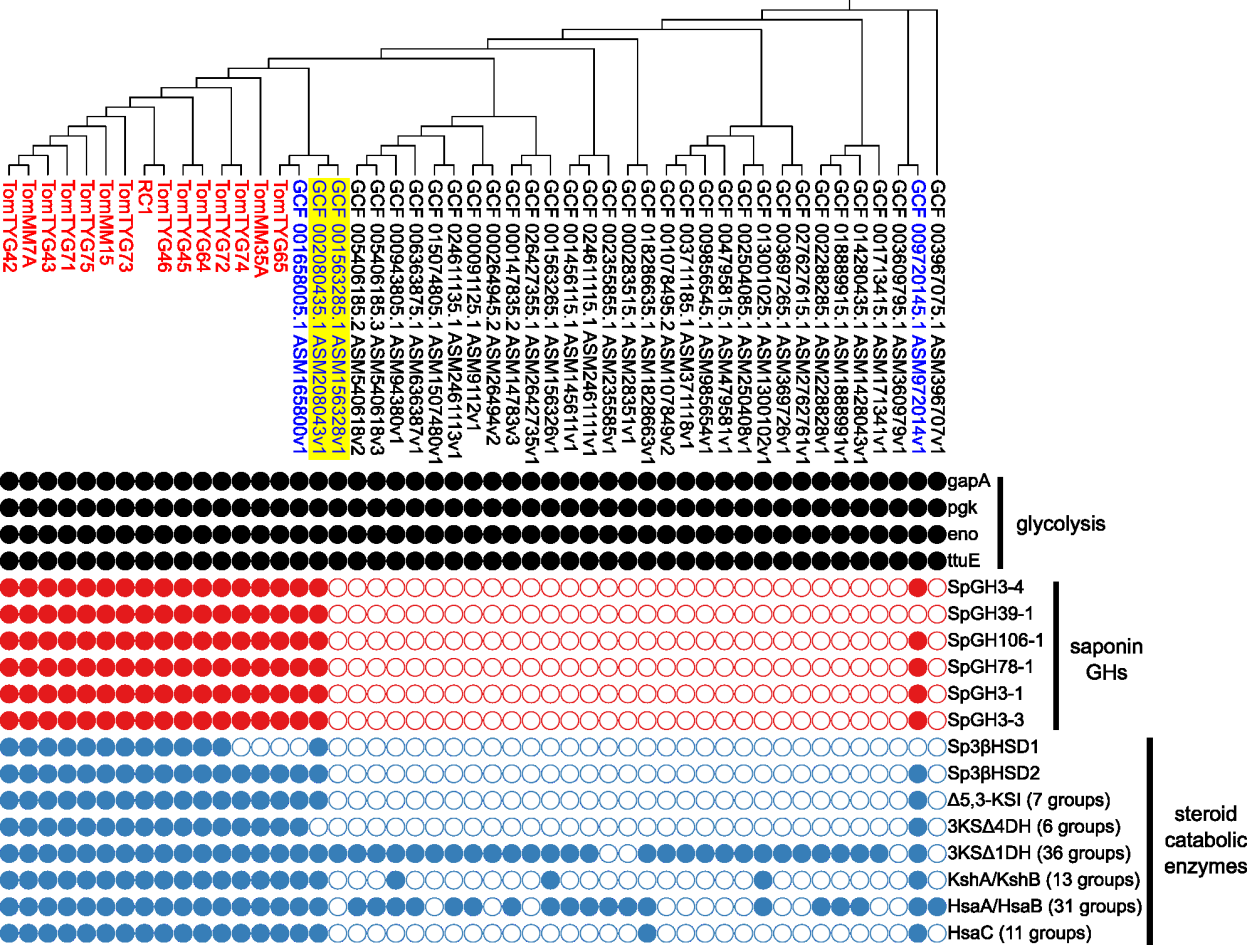
Phylogenetic distribution of the genes encoding saponin glycoside hydrolases (GHs) and steroid-catabolizing enzymes in *Sphingobium* spp. including α-tomatine-degrading isolates. Gene presence and absence are indicated by closed and open circles, respectively. The number of groups in parentheses represents the number of groups of orthologous genes (OGs) annotated for each enzyme, and enzymes with at least one OG are indicated by closed circles. The red and blue letters indicate our isolates and strains from the public database mentioned in this article, respectively. GCF 002080435.1 ASM208043v1 and GCF 001563285.1 ASM156328v1 highlighted in yellow correspond to NBRC16415 and JCM17233 in Fig. 6, respectively.

We identified several steroid-catabolizing enzymes and saponin GHs in RC1. Subsequently, we investigated whether the genes encoding these enzymes were present in the genus *Sphingobium*, including other α-tomatine-degrading isolates. We performed a comparative genomic analysis between 15 of our isolates and 34 strains that have whole-genome sequences that are registered in public databases (Fig. 5; Data Set S4). A phylogenetic tree constructed based on core genes showed that our α-tomatine-degrading isolates were located in a specific clade (Fig. 5). All α-tomatine-degrading isolates possessed a set of homologous genes encoding Sp3βHSD2, steroid-catabolizing enzymes, and six saponin GHs (Fig. 5). Unlike *Sp3βHSD2*, *Sp3βHSD1* was absent in TomTYG74, TomTYG65, and TomMM35A; however, this probably did not affect the degradation of relevant saponins because of complementation by *Sp3βHSD2* (Fig. 5). Regarding the strains from the public database, GCF_001658005.1 possessed the gene set described above, with the exception of *Sp3βHSD1*, suggesting that it can completely degrade steroid-type saponins (Fig. 5). In contrast, because 3KSΔ4DH and SpGH39-1 were absent in GCF_002080435.1 and GCF_009720145.1, respectively, the two strains may partially degrade steroid-type saponins and then accumulate the precursors for each enzyme as the degradation intermediates (Fig. 5). The strains from the public database, with the exception of GCF_001658005.1, GCF_002080435.1, and GCF_009720145.1, did not possess saponin GHs or most of the steroid-catabolizing enzymes, suggesting that they are unable to degrade steroid-type saponins (Fig. 5).

**Fig 6:**
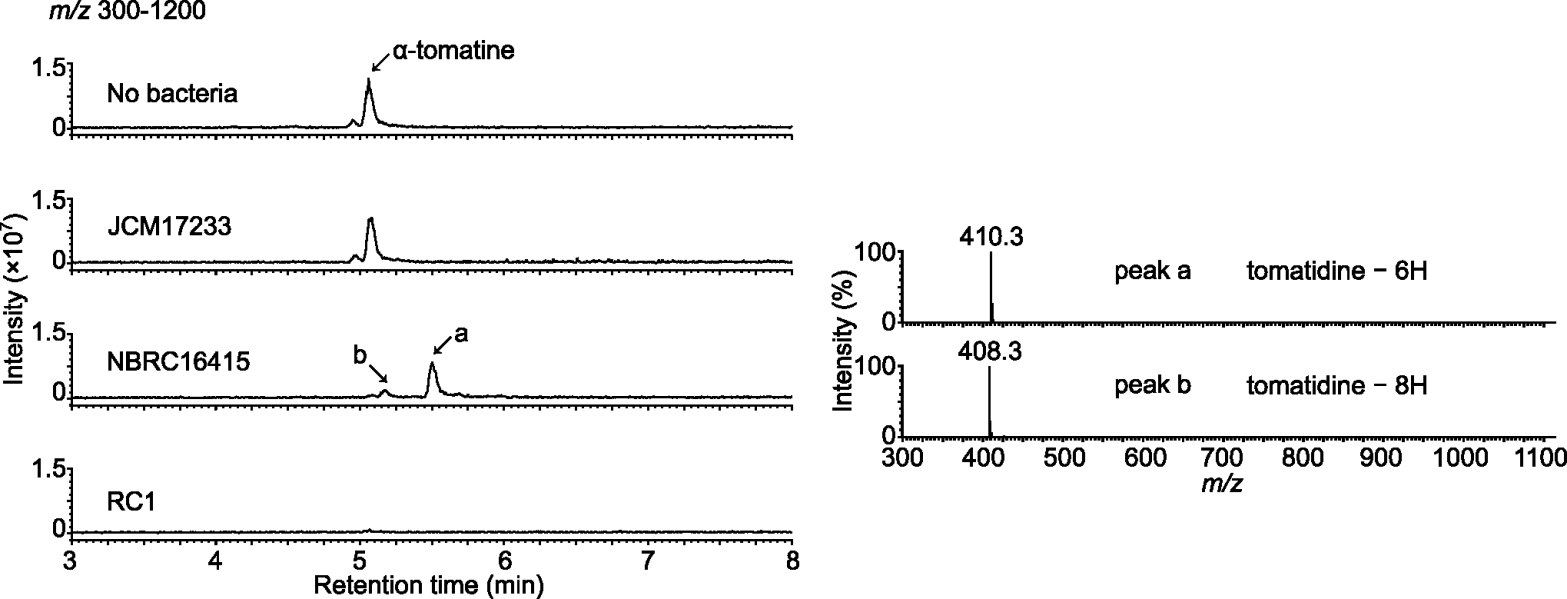
α-Tomatine-degradation activities in three *Sphingobium* strains, JCM17233, NBRC16415, and RC1. LC-MS analysis of the reaction products obtained from the respective resting cells incubated for 24 h using α-tomatine as a substrate. A reaction mixture without cells was used as the negative control (no bacteria). Representative data of the degradation activities measured in technical triplicates are shown. The total ion current chromatogram obtained in the positive ionization mode with a full-scan range of *m/z* 300–1200 is shown. The mass spectra of peaks a and b (reaction products), as indicated by arrows in the chromatogram, are shown. NBRC16415 and JCM17233 correspond to GCF 002080435.1 ASM208043v1 and GCF 001563285.1 ASM156328v1 in Fig. 5, respectively.

Next, we measured the degradation activities of two *Sphingobium* strains, *S. herbicidovorans* MH (NBRC16415) and *Sphingobium* sp. MI1205 (JCM17233), corresponding to GCF_002080435.1 and GCF_001563285.1, respectively (Fig. 6), which were closely related to each other (Fig. 5). Incubation of their resting cells with α-tomatine showed that NBRC16415, which carried a gene set that excluded *3KSΔ4DH*, degraded this compound and produced two peaks with retention times of 5.7 and 5.4 min and major mass fragment ions at *m/z* 410 and *m/z* 408, respectively (Fig. 6), which were thought to be putative degradation intermediates. In contrast, JCM17233, which carried only *3KSΔ1DH* in its genome, did not degrade α-tomatine (Fig. 6). These results revealed that the presence of genes encoding degradative enzymes was consistent with the degradation activities of saponins.

## DISCUSSION

In this study, we identified the enzymes that were responsible for the degradation of α-tomatine and related steroidal saponins in *Sphingobium* sp. RC1, which was isolated from tomato roots. SpGH3-4, SpGH39-1, and SpGH3-1/-3 coordinately degraded α-tomatine to tomatidine. The reported GH3 hydroxylase family of microbial saponin degradation enzymes exhibits 1,2-, 1-4-, and 1-6-β-d-glucosidase and 1-6-α-l-arabinopyranosidase activities (34, 36, 50 – 52). Avenacinase in the oat fungal pathogen *Gaeumannomyces graminis* var. *avenae* removes the terminal β-1,2- and β-1,4-linked d-glucose molecules from avenacin A-1 (34). In turn, tomatinase of the tomato pathogen *Septoria lycopersici* removes the terminal β-1,2-linked d-glucose from α-tomatine, which is named β_2_-tomatinase because it yields β_2_-tomatine as the reaction product (36). Although the two GHs specifically recognize their respective host saponins as substrates, both GHs belong to the GH3 family and have 1,2-β-d-glucosidase activity for their substrates (50). GH3 enzymes with other activities, for example 1,6-β-d-glucosidase and 1-6-α-l-arabinopyranosidase activities, have also been identified as ginsenosidases in several microorganisms (51, 52). Here, none of the GH3 enzymes tested in *Sphingobium* sp. RC1 exhibited 1,2-β-d-glucosidase activity toward α-tomatine, whereas SpGH3-1, SpGH3-3, and SpGH3-4 displayed different activities (Fig. 3; Fig. S7 and S8). These findings indicated that microbial saponin-hydrolyzing GH3 enzymes possess diverse substrate specificities and regioselectivities.

Alternatively, a gene product detected in RC1, SpGH39-1, catalyzed 1,2-β-d-glucosidase activity toward β_1_-tomatine. Thus, it may have acted as a multifunctional enzyme that removed both β-1,2-d-glucose and β-1,3-d-xylose from α-tomatine, to produce γ-tomatine (Fig. 3). Most of the bacterial GH39 enzymes characterized to date exhibit β-xylosidase activity (53). The GH39 enzymes in *Thermoanaerobacterium thermosaccharolyticum* and *Sphingomonas* sp. JB13 have been identified as ginsenosidases that hydrolyze the C-6 outer β-1,2-d-xylosidic linkage (54, 55). In contrast, a GH39 enzyme in *Croceicoccus marinus* E4A9 (CmGH1) had β-glucosidase activity, instead of β-d-xylosidase activity (56). Future biochemical analyses of GH39 enzymes, including SpGH39-1, will provide new insights into their multifunctional catalytic mechanism.

GH78 enzymes are found in bacteria and fungi, whereas GH106 enzymes are found exclusively in bacteria. All characterized enzymes in both families predominantly exhibited α-l-rhamnosidase activities. SpGH106-1 and SpGH78-1 showed putative 1,2- and 1,4-α-l-rhamnosidase activities toward steroidal saponins, respectively (Fig. S7 and S8). A GH78 enzyme present in the *Absidia* sp. 39 fungal strain has been identified as a ginsenosidase that hydrolyzes the C-6 outer α-1,2-l-rhamnosidic linkage (57). Recently, in two bacterial strains, i.e., *Arthrobacter* sp. S41 from soils surrounding green potato peels and *Glutamicibacter halophytocola* S2 from the gut of a potato pest (*Phthorimaea operculella*), three GHs belonging to the GH2, GH3, and GH78 families, respectively, were reported to exist as the gene cluster in respective genomes and to catalyze the complete deglycosylation of both α-chaconine and α-solanine (58, 59). All three GHs from the latter strain have been characterized as multifunctional enzymes that cleave multiple types of glycosidic bonds (59). In contrast, SpGH78-1 did not recognize α-solanine as a substrate (Table S3), indicating that its enzymatic properties are different from those of the GH78 enzymes characterized previously. A GH106 enzyme responsible for saponin degradation has not been identified to date. Therefore, SpGH106-1 was an enzyme with saponin-hydrolyzing ability that was unique in this family.

Microbial steroid-degrading enzymes have been well studied in several soil-borne microorganisms, and degradation pathways have been proposed (39). Metagenomics analyses of the genes encoding steroid-catabolizing enzymes from various environments revealed that those isolated from Alphaproteobacteria and Actinobacteria are predominant in the rhizosphere, and that the former mainly consist of *Sphingomonadaceae* and Rhizobiales (60). In general, plants produce a complex mixture of sterols, which are a subgroup of steroids that serve as integral components of the lipid bilayer of biological membranes and the precursors of plant hormones in the brassinosteroid class (61, 62), implying that the ability of those microbial taxa to degrade steroids contributes to their utilization as nutrients and colonization in the plant rhizosphere. Accordingly, several strains of *Mycobacteria* and *Rhodococcus* from Actinobacteria and *Novosphingobium* and *Sphingomonas* from *Sphingomonadaceae* can degrade steroids, such as cholesterol, cholic acids, androgens, estrogens, and their derivatives, to use them as energy sources (63 – 66). The early steps of steroid degradation, i.e., C3 oxidation and C1,4-desaturation (67), have also been observed in tomatidine modification by *Nocardia* and *Arthrobacter* of Actinobacteria, which convert it into tomatid-4-en-3-one (68, 69). Here, Sp3βHSD1 and Sp3βHSD2 of *Sphingobium* sp. RC1, which are the orthologs of steroid-degrading enzymes, metabolized tomatidine, solanidine, and diosgenin to produce peaks with mass fragments corresponding to their respective putative 4-en-3-one derivatives (Fig. 4; Fig. S12 and S13). Based on the known steroid degradation pathway (Fig. S5), we proposed a putative tomatidine degradation pathway in RC1 (Fig. S16). The identification of the enzymes that catalyze the first step of sapogenin degradation may pave the way toward the characterization of the saponin degradation pathway in soil bacteria, because we can postulate that orthologous genes to the steroid degradation pathway are involved in the sapogenin degradation pathway. This was supported by our observation that NBRC16415, with a gene set that excluded 3KSΔ4DH, did not completely degrade tomatidine and metabolized α-tomatine to putative degradation intermediates (Fig. 6).

The enzymatic degradation of saponins contributes to the pathogenesis of various microorganisms. For instance, oat, tomato, and potato pathogens produce GHs termed avenacinase, tomatinase, and α-chaconinase, which remove the sugar moiety from the avenacin A-1, α-tomatine, and α-chaconine accumulated in each host plant, respectively (34 – 38). Avenacinase-deficient mutants of the oat fungal pathogen *Gaeumannomyces graminis* var. *avenae* are unable to infect oats (70). Moreover, the heterologous expression of the tomatinase gene from the tomato fungal pathogen *Septoria lycopersici* in another tomato fungal pathogen (*Cladosporium fulvum*) enhanced its virulence toward tomato, and a tomatinase-deficient mutant of *C. fulvum* exhibited reduced virulence (49, 71). Accordingly, the RC1 α-tomatine-degrading enzymes identified in this study may also be involved in interactions with tomato plants.

Our comparative genomics analysis revealed that the orthologs of the degradation enzymes were present not only in our α-tomatine-degrading isolates belonging to a phylogenetically specific clade of *Sphingobium*, but also in GCF_009720145.1, which is phylogenetically distant from them (Fig. 5). In the RC1 genome, *SpGH3-4*, *SpGH39-1*, and *SpGH3-3* were present in a chromosome, whereas *SpGH3-1*, *SpGH106-1*, and *SpGH78-1* were located in a plasmid (Fig. S4). These data imply that both vertical (inherited from a common ancestor) and horizontal (transferred from a phylogenetically unrelated organism of the same generation) gene transmission events resulted in the acquisition of the genes encoding degradation enzymes in some strains of *Sphingobium*. This was consistent with the presence of genomic features in *Arthrobacter*, in which catabolic genes for nicotine and santhopine, which are PSMs secreted from tobacco roots, indicate their transfer in both vertical and horizontal manners (72). Because bacterial isolates that metabolize PSMs are isolated from the rhizosphere of the plant species that accumulate these PSMs (73, 74), we proposed that the catabolic abilities of *Arthrobacter* are associated with their ability to colonize tobacco roots (72). These observations support the hypothesis that the genes involved in saponin degradation in tomato-root-associated *Sphingobium* strains provide them with a survival advantage in the presence of bioactive saponins in the tomato rhizosphere.

## MATERIALS AND METHODS

### Chemicals, plant materials, bacterial strains, and soils

α-Tomatine, diosgenin, glycyrrhizin, glycyrrhetic acid, pregnenolone, 5α-pregnane-3,20-dione, and progesterone were purchased from Tokyo Chemical Industry Co., Ltd. (Tokyo, Japan). Tomatidine and dioscin were purchased from Cayman Chemical (Ann Arbor, MI, USA), and 2,6-dichlorophenol indophenol (DCPIP) and β-nicotinamide-adenine dinucleotide oxidized form (β-NAD^+^) were purchased from Nakalai Tesque, Inc. (Kyoto, Japan). α-Solanine, solanidine, a mixture of soyasaponin from soybeans (mainly soyasaponin Bb), soyasapogenol B, and isopregnanolone were purchased from Extrasynthese (Genay, France), Sigma-Aldrich (St. Louis, MO, USA), FUJIFILM Wako Pure Chemical Corporation (Osaka, Japan), Tokiwa Phytochemical (Chiba, Japan), and Santa Cruz Biotechnology (Dallas, TX, USA), respectively. Seeds of tomato (*S. lycopersicum*) cv. Benisuzume were purchased from the Institute for Horticultural Plant Breeding (Chiba, Japan). The bacterial strains used in this study are listed in Table S1. The *S. herbicidovorans* MH (NBRC16415) and *Sphingobium* sp. MI1205 (JCM17233) strains were provided by the Biological Resource Center, NITE (NBRC) (Chiba, Japan) and Japan Collection of Microorganisms, RIKEN BRC (Ibaraki, Japan) through the National BioResource Project of the MEXT, respectively. Field soils were sampled at the field of the Kyoto University of Advanced Science (KUAS), Kameoka, Kyoto, Japan (34°59′37.7″N, 135°33′05.0″E), air dried, and sieved as described in our previous study (75).

### Cultivation of field-grown tomato plants and sampling of their roots

Tomato seeds were sown in pots filled with a 1:1 mixture of vermiculite and field soils. Seedlings were grown in the laboratory for 4 weeks at 25°C under a 16-h light/8-h dark cycle, and then in a greenhouse for 10 weeks. They were planted in the field of KUAS on 12 June 2020. The roots of the tomato plants were sampled at the flowering stage on 9 July 2020. They were kept cool using an ice pack and transported to the laboratory. The rhizosphere and rhizoplane soils were removed from the roots via gentle shaking for 5 min and sonication for 5 min in phosphate-buffered saline (PBS; pH 7.0) containing 130 mM NaCl, 7 mM Na_2_HPO_4_, 3 mM NaH_2_PO_4_, and 0.02% Silwet L-77 (76). After rinsing with tap water, the endosphere compartments were stored at 4°C until bacteria were isolated (one overnight).

### Isolation of *Sphingobium* spp. from α-tomatine-treated soils and field-grown tomato plants

The isolation of bacterial strains from α-tomatine-treated soils was carried out as described in our previous work (28), with minor modifications. Briefly, the soil suspensions diluted with sterile water were distributed onto agar plates prepared using tryptone yeast extract glucose (TYG) medium (77), in addition to mineral salt buffer (MS) medium (78) containing 20 µg mL^−1^ of α-tomatine or tomatidine as the sole carbon source. To isolate tomato-root-inhabiting bacteria, 1 g of the endosphere compartments was homogenized with a mortar and pestle in 10 mL of 10 mM MgCl_2_ solution and distributed onto TYG agar plates. All plates were incubated for up to 7 days at 28°C. Yellow colonies were picked up from the plates, and their genomic DNA was extracted using the hot-alkaline DNA extraction method; each colony was suspended in 10 µL of a buffer containing 25 mM NaOH and 0.2 mM EDTA and incubated at 95°C for 30 min, followed by the addition of 10 µL of 40 mM Tris-HCl solution (pH 6.8). Using these DNA extracts as templates, the 16S rRNA genes in the respective isolates were PCR amplified using KOD FX Neo (TOYOBO, Osaka, Japan) and the primer set: 10F (5′-GTTTGATCCTGGCTCA-3′) and 800R (5′-TACCAGGGTATCTAATCC-3′). The PCR conditions were as follows: 94°C for 2 min; followed by 35 cycles at 98°C for 10 s, 50°C for 30 s, and 68°C for 1 min. The PCR products were purified using a Wizard Genomic DNA Purification Kit (Promega, Madison, WI, USA), according to the manufacturer’s instructions, and sequenced using the 10F primer. ClustalW was used to align the obtained 16S rRNA V4 region. Bacterial isolates that were annotated as *Sphingobium* spp. by a BLAST search were cultured in a growth medium (pH 7.0) containing 10 g L^−1^ peptone, 10 g L^−1^ beef extract, and 5 g L^−1^ NaCl, and then stored in a 20% glycerol solution at −80°C.

### Genomic DNA extraction, whole-genome sequencing, and annotation

Each stock culture of bacterial isolates was streaked onto the agar plates. A single colony was pre-cultured in 2 mL of the growth medium and further cultivated in 10 mL of the same medium for 2 days at 28°C, respectively. The culture was harvested by centrifugation at 4,000 × *g* for 5 min. The cell pellets were washed with 5 mL of TE buffer consisting of 10 mM Tris-HCl (pH 8.0) and 1 mM EDTA, and stored at −30°C until genomic DNA extraction. Genomic DNA was extracted as described previously, with modifications (79). The cell pellets were lysed in 600 µL of TE buffer containing 20 mg mL^−1^ lysozyme and incubated for 30 min at 37°C. Fifty microliter of 20% (wt/vol) sodium dodecyl sulfate in aqueous solution and 25 µL of TE buffer containing 20 mg mL^−1^ proteinase K were added to the cells, followed by incubation for 30 min at 37°C. The cleared lysate was forced through a syringe (38 × 0.8 mm) 10 times, and proteins were removed using the TE-saturated phenol:chloroform:isoamyl alcohol (25:24:1) method. The genomic DNA was collected using the ethanol precipitation method and dissolved in 100 µL of TE buffer. The DNA concentration was measured using a BioSpec-nano instrument (Shimadzu, Kyoto, Japan) and a Qubit 2.0 Fluorometer (Thermo Fisher Scientific, Waltham, MA, USA).

Library construction and whole-genome sequencing were performed as described previously, with minor modifications (72). Briefly, a DNA library was cut off at 15 kbp using the Blue Pippin size-selection system (Sage Science, Beverly, MA, USA). Genomes were assembled using the Hierarchical Genome Assembly Process v.4 within SMRTlink (v.10.0 for RC1; v.8.0 for the remaining strains), and exhibited the expected size. Circlator v.1.5.5 (80) was used to evaluate whether the genome assemblies were circularizable and to predict the location of the starting position.

The obtained genomes were automatically annotated using the Prokka pipeline, to predict CDSs, tRNAs, and rRNAs (43). The completeness and redundancy of genomic data were evaluated via the assignment of the protein sequences in each isolate to the sphingomonadales_odb10 data set using BUSCO, version v5.4.4 (42). KEGG orthologs were assigned to proteins in RC1 using Kofam KOALA (81). Finally, CAZymes (including GHs) in RC1 were extracted from the RC1 total proteins using dbCAN2 with default parameters (44).

### Measurements of the saponin- and sapogenin-degrading activities using resting cells

The saponin- and sapogenin-degrading activities of the bacterial strains were measured using their resting cells. Each strain was cultivated in 2 mL of the growth medium for 2 days, followed by centrifugation of the culture at 4,000 × *g* for 5 min. Cell pellets were washed twice with 1 mL of MS medium and then resuspended to OD_600_ = 2.0 using the same medium. A resting cell reaction was performed in 100 µL of MS medium including 20 µM each substrate and cell suspensions at OD_600_ = 1.0. The reaction was carried out at 28°C for 3 or 24 h, and stopped using 100 µL of methanol. The reaction mixture was centrifuged at 10,000 × *g* for 1 min, filtered through a 0.45 µm Minisart RC4 filter (Sartorius, Göttingen, Germany), and applied to LC-MS analysis.

### Heterologous expression of recombinant proteins in *E. coli*

The coding sequences of saponin-degrading candidate genes were PCR amplified using the RC1 genomic DNA as a template, which was extracted using the hot-alkaline DNA extraction method described above with the primer sets listed on Data Set S3. The amplified DNA fragments were ligated into the pGEM-T Easy Vector (Promega). The DNA fragments of CDS were ligated into pET22b (Merck KGaA, Darmstadt, Germany) using restriction enzymes or an In-Fusion HD Cloning Kit (Takara Bio, Shiga, Japan). In addition, the CDSs of *Sp3KSΔ4DH1* were codon-optimized for *E. coli* expression using the Codon Optimization Tool (ExpOptimizer) (https://novoprolabs.com/tools/codon optimization) provided by NovoPro Inc. (Shanghai, China). The codon-optimized sequences with a TIR-2 sequence located upstream, which is a promoter that can increase the protein-production yield in *E. coli* (82), were synthesized and inserted into pET28a by Twist Bioscience (South San Francisco, CA, USA). *E. coli* strain BL21 (DE3) (Takara Bio) was transformed with the constructed vector and grown at 37°C in 50 mL of lysogeny broth containing 50 µg mL^−1^ ampicillin until its OD_600_ reached 0.5. Recombinant protein expression was induced by adding isopropyl β-d-1-thiogalactopyranoside at a final concentration of 100 µM and was continued for 20 h at 18°C. The culture was harvested by centrifugation at 10,000 × *g* for 5 min at 4°C. The cell pellets were washed twice with cold PBS buffer and stored at −30°C until the *in vitro* assay. Each cell pellet was resuspended in 1 mL of a cold lysis buffer consisting of 50 mM sodium phosphate (pH 8.0), 300 mM NaCl, and 10 mM imidazole; followed by five rounds of sonication for 15 s using an ultrasonic homogenizer (Sonifier Model 250A; Branson, Danbury, CT, USA) with the following settings: a duty cycle of 50% and an output control of 20%. The homogenate was centrifuged at 10,000 × *g* for 5 min at 4°C, and the supernatant was used for the purification of His-tagged proteins. One hundred and fifty microliters of Ni-NTA agarose (QIAGEN) were loaded onto Micro Bio-Spin Chromatography Columns (bed volume, 0.8 mL) (Bio-Rad, Hercules, CA, USA). The resin was equilibrated with 600 µL of the lysis buffer and centrifuged at 1,000 × *g* for 1 min at 4°C, to remove the buffer. His-tagged proteins in 600 µL of the supernatant were bound to the Ni-NTA resin and washed twice with 600 µL of a wash buffer consisting of 50 mM sodium phosphate (pH 8.0), 300 mM NaCl, and 20 mM imidazole. The adsorbed proteins were eluted twice with 100 µL of an elution buffer consisting of 50 mM sodium phosphate (pH 8.0), 300 mM NaCl, and 250 mM imidazole. The concentration of the purified recombinant proteins was measured using a Qubit 2.0 Fluorometer and were used in *in vitro* assays.

### Measurements of saponin- and sapogenin-degrading activities using recombinant proteins

An *in vitro* assay of recombinant Sp3βHSD1 and Sp3βHSD2 was performed using 100 µL of a reaction mixture consisting of 50 mM sodium phosphate (pH 7.0), 1 mM β-NAD^+^ (as a coenzyme), 50 µM sapogenins and pregnane derivatives (as substrates), and 1 µg of each recombinant protein. The enzymatic activities of Sp3KSΔ4DH1 were measured by adding 1 µg of each recombinant protein to the reaction mixtures described above. For the latter, 1 mM DCPIP was added as an electron acceptor. An *in vitro* assay of recombinant GHs was performed using 100 µL of a reaction mixture consisting of 50 mM potassium phosphate (pH 6.0), 50 µM saponins (as substrates), and 1 µg of the respective recombinant proteins. All reactions were carried out at 37°C for 2 h, and then stopped by adding 100 µL of *n*-butanol saturated with water. The reaction mixture was extracted three times by vortexing and centrifugation at 10,000 × *g* for 1 min. The organic phase was collected and dried *in vacuo*. The residue was dissolved in 200 µL of methanol, filtered through a 0.45-µm Minisart RC4 filter (Sartorius), and applied to LC-MS analysis.

### LC-MS analysis of reaction products using resting cells and recombinant proteins

The microbial and enzymatic reaction products were analyzed using an Acquity ultra-high-performance liquid chromatography (UPLC) HClass/Xevo TQD instrument (Waters). For each sample, 2 µL was injected into an Acquity UPLC HSS T3 column (1.7 µm; 2.1 × 100 mm^2^; Waters) using a UPLC HSS T3 VanGuard Precolumn (1.7 µm, 2.1 × 5 mm^2^) or an Acquity UPLC BEH C18 column (1.7 µm, 2.1 × 50 mm^2^; Waters) with an UPLC BEH C18 VanGuard Precolumn (1.7 µm; 2.1 × 5 mm^2^). The former was used for the analysis of the reaction products from resting cells of *Sphingobium* isolates with α-tomatine (Fig. S1), followed by recombinant GHs with α-tomatine and α-solanine (Fig. S7 and S8); whereas the latter was used for the remaining ones. The column oven temperature was set at 40°C. The mobile phases consisted in water containing 0.1% (vol/vol) formic acid (solvent A) and acetonitrile (solvent B). The flow rate was set at 0.2 mL min^−1^. The mass spectra were obtained in the positive electrospray ionization mode using following settings: cone voltage of 30 V; capillary voltage of 3.15 kV; source temperature of 150°C; desolvation gas temperature of 400°C; nebulizer and desolvation N_2_ gas flow rates of 50 and 800 L h^−1^, respectively. The elution programs and mass conditions used for each analysis are described in Text S1. The data obtained were analyzed using the MassLynx v. 4.1 software (Waters).

### Comparative genomic analysis of *Sphingobium* spp.

A comparative genomic analysis of *Sphingobium* spp. was performed on our isolates and on with complete genome sequences that were registered in NCBI database, as listed in Data Set S4. Their coding sequences, which were annotated by the Prokka pipeline, as described above, were used to cluster orthologous genes (OGs) via the Roary v3.13.0 pipeline with a minimum percentage identity of 75% for BLASTP (83). Genes that were present in 95% or more of the strains were defined as core genes. All core genes were aligned using MAFFT (84), and the alignment was employed to build a phylogenetic tree using FastTree (85). The binary matrix with presence and absence of genes across all strains was used to draw the phylogenetic distribution of metabolic genes using Interactive Tree Of Life (iTOL), which is an online tool for the display, manipulation, and annotation of phylogenetic trees (86).

### Total RNA extraction, RNA-seq, and transcriptome analysis

RC1 cell suspensions at OD_600_ = 0.5 in MS medium (7.5 mL) were treated with 75 µL of 5 mM α-tomatine dissolved in methanol. An equal volume of methanol was used as the mock treatment. Both α-tomatine- and mock-treatments were performed in technical duplicates. After incubation at 28°C for 3 h, 1 mL of the suspension was centrifuged at 10,000 × *g* for 1 min, and cell pellets were stored at −80°C until total RNA extraction. The residual suspension was used to investigate the induction of α-tomatine-degrading activity by α-tomatine treatment. The resting cells were prepared as a substrate as described in the “Measurements of the saponin- and sapogenin-degrading activities using resting cells” subsection. The resting cell reaction was performed in 1 mL of MS medium consisting of 50 µM α-tomatine and cell suspensions at OD_600_ = 0.5. After 1, 2, and 3 h from the start of the reaction, 100 µL of each of the suspensions was collected and mixed with an equal volume of methanol and applied to LC-MS analysis, as described above.

Total RNA was extracted from RC1 cells treated with α-tomatine using the TRI reagent (Cosmo Bio Co., Ltd., Tokyo, Japan), according to the manufacturer’s instructions. The RNA concentration was measured using a Qubit 2.0 Fluorometer. The total RNA library was prepared using the TruSeq stranded total RNA library (bacteria) (Illumina, San Diego, CA, USA) and applied to 2 × 100 bp paired-end sequencing on a NovaSeq6000 platform (Illumina) at Macrogen Japan Corp. (Tokyo, Japan). The paired-end reads were used to quantify the abundances of transcripts with CDSs of RC1 (as a reference) using Kallisto version 0.46.2 (87), which is an RNA-seq quantification program, with default parameters. The obtained Kallisto pseudo-counts (TPM) are listed in Table S2 and Data Set S5.

## Data Availability

The acquired sequence data sets that supported the conclusions of this study were registered in the DNA Data Bank of Japan (DDBJ) Sequence Read Archive (accession numbers: DRA015721 and DRA015722 for the whole-genome sequencing of *Sphingobium* isolates and transcriptome of RC1, respectively). The nucleotide sequences of *SpGH3-1*, *SpGH3-3*, *SpGH3-4*, *SpGH39-1*, *SpGH78-1*, *SpGH106-1*, *Sp3βHSD1*, *Sp3βHSD2*, and *Sp3KSΔ4DH1*, which were identified in this study, were registered to DDBJ (accession numbers: LC754524, LC754525, LC754526, LC754527, LC754528, LC754529, LC754530, LC754531, and LC754532, respectively).

## SUPPLEMENTAL MATERIAL

DATA SET S1 mbio.00599-23-s0001.xlsx Assembly results of the *Sphingobium* isolates used in this study.

DATA SET S2 mbio.00599-23-s0002.xlsx List of the RC1 glycoside hydrolase genes annotated using dbCAN2.

DATA SET S3 mbio.00599-23-s0003.xlsx Primer sequences used in this study.

DATA SET S4 mbio.00599-23-s0004.xlsx Public genome assembly used in the comparative genomic analysis.

DATA SET S5 mbio.00599-23-s0005.xlsx The transcript levels of all genes in α-tomatine- and mock-treated RC1 cells.

TEXT S1 mbio.00599-23-s0006.docx Elution programs and mass conditions for detection of each reaction product in LC-MS analysis.

Supplemental Material mbio.00599-23-s0007.pdf Fig. S1 to S16; Tables S1 to S3.

ASM does not own the copyrights to Supplemental Material that may be linked to, or accessed through, an article. The authors have granted ASM a non-exclusive, world-wide license to publish the Supplemental Material files. Please contact the corresponding author directly for reuse.
