# Supplementary material for: Tomato root-associated Sphingobium harbors genes for catabolizing toxic steroidal glycoalkaloids
Source: mBio. 2023 Sep 29;14(5):e00599-23. doi: 10.1128/mbio.00599-23 (PMC10653915; doi:10.1128/mbio.00599-23)
Supplement: TEXT S1 — Elution programs and mass conditions for detection of each reaction product in LC-MS analysis. [file mbio.00599-23-s0006.docx]

**Supplemental methods**

**TEXT S1.** Elution programs and mass conditions for detection of each reaction product in LC-MS analysis.

In all analyses, the mobile phases consisted in water containing 0.1% (v/v) formic acid (solvent A) and acetonitrile (solvent B). Elution programs and mass conditions for each LC-MS analysis is as follows.

***Reaction products obtained from resting cells using α-tomatine (Fig. S1)***

The elution program was 10%–55% B from 0 to 15 min (linear gradient), 55% B from 15 to 17.5 min, 100% B from 17.5 to 22.5 min, and 10% B from 22.5 to 28.5 min. The total ion current chromatogram was obtained in the positive ionization mode with a full-scan range of *m/z* 300–1400.

***Reaction products obtained from resting cells using α-tomatine, tomatidine, α-solanine, and solanidine (Figs 2, 6, S2, and S6)***

The elution program was 10%–90% B from 0 to 10 min (linear gradient), 90% B from 10 to 12 min, 100% B from 12 to 16 min, and 10% B from 16 to 21 min. The total ion current chromatogram was obtained in the positive ionization mode with a full-scan range of *m/z* 250–500 for tomatidine and solanidine, and of *m/z* 300–1200 for α-tomatine and α-solanine (as substrates), respectively.

***Reaction products obtained from recombinant enzymes using pregnane derivatives as substrates (Figs S9–11)***

The elution program was 10%–75% B from 0 to 10 min (linear gradient), 75%–100% B from 10 to 12 min (linear gradient), 100% B from 12 to 16 min, and 10% B from 16 to 21 min. The total ion current chromatogram was obtained in the positive ionization mode with a full-scan range of *m/z* 250–500.

***Reaction products obtained from recombinant enzymes using tomatidine and solanidine as substrates (Figs 4 and S12).***

The elution program was 10%–55% B from 0 to 10 min (linear gradient), 55% B from 10 to 12 min, 100% B from 12 to 16 min, and 10% B from 16 to 21 min. The total ion current chromatogram was obtained in the positive ionization mode with a full-scan range of *m/z* 250–500.

***Reaction products obtained from resting cells or recombinant enzymes using diosgenin and dioscin as substrates (Figs 2, S8, and S13; Table S3)***

The elution program was 10%–100% B from 0 to 10 min (linear gradient), 100% B from 10 to 14 min, and 10% B from 14 to 19 min. The total ion current chromatogram was obtained in the positive ionization mode with a full-scan range of *m/z* 250–500 for diosgenin and *m/z* 300–1200 for dioscin (as substrates), respectively.

***Reaction products obtained from resting cells or recombinant enzymes using glycyrrhetic acid,*** ***glycyrrhizin, soyasaponin Bb, and soyasapogenol B as substrates (Figs 2 and S14–15; Table S3).***

The elution program was 10%–90% B from 0 to 10 min (linear gradient), 90%–100% B from 10 to 12 min (linear gradient), 100% B from 12 to 16 min, and 10% B from 16 to 21 min. The total ion current chromatogram was obtained in the positive ionization mode with a full-scan range of *m/z* 250–500 for glycyrrhetic acid and soyasapogenol B and *m/z* 300–1200 for glycyrrhizin and soyasaponin Bb (as substrates), respectively.

***Reaction products obtained from recombinant enzymes using α-tomatine and α-solanine as substrates (Figs 3 and S7; Table S3)***

The elution program was 5%–35% B from 0 to 20 min (linear gradient), 35%–55% B from 20 to 25 min (linear gradient), 55% B from 25 to 27 min, 100% B from 27 to 32 min, and 10% B from 32 to 38 min. The total ion current chromatogram was obtained in the positive ionization mode with a full-scan range of *m/z* 300–1200.
