# Supplementary material for: Tomato root-associated Sphingobium harbors genes for catabolizing toxic steroidal glycoalkaloids
Source: mBio. 2023 Sep 29;14(5):e00599-23. doi: 10.1128/mbio.00599-23 (PMC10653915; doi:10.1128/mbio.00599-23)
Supplement: Supplemental Material — Fig. S1 to S16; Tables S1 to S3. [file mbio.00599-23-s0007.pdf]

FIG S1

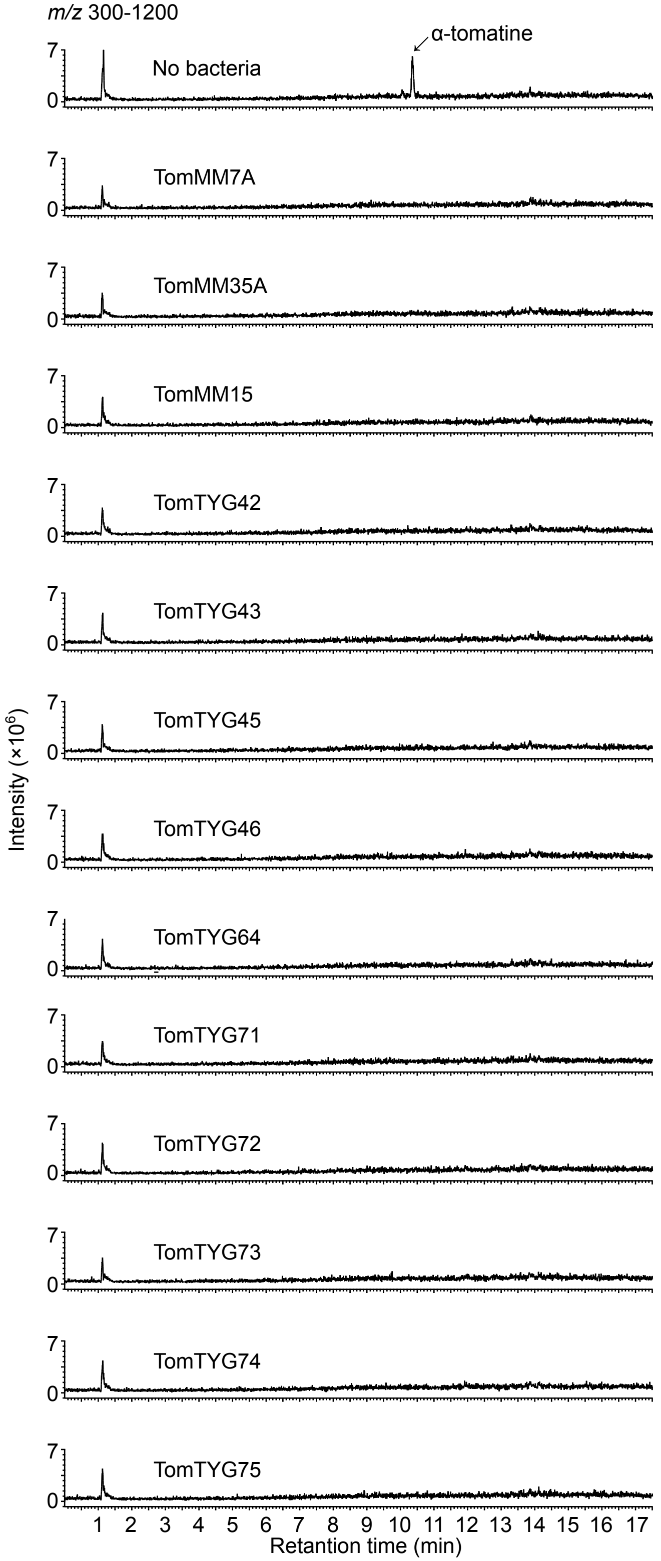

FIG S1. α-Tomatine-degradation activities in *Sphingobium* isolates. LC-MS analysis of the reaction products from the respective resting cells incubated for 24 h using α-tomatine as a substrate. A reaction mixture without any strains was used as the negative control (no bacteria). The degradation activities measured in one replicate (n =1) are shown. The total ion current chromatogram obtained in the positive ionization mode with a full-scan range of *m/z* 300–1400 is shown.

FIG S2

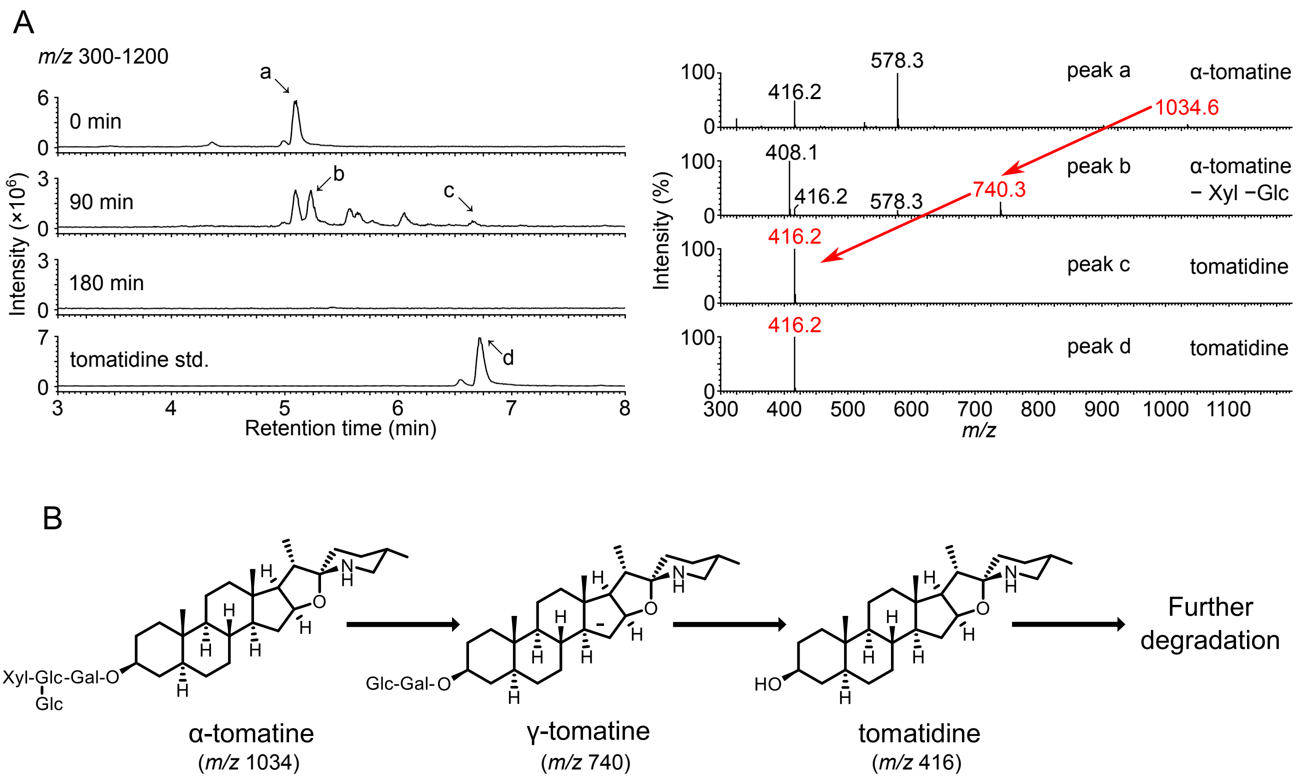

FIG S2. α-Tomatine degradation products in RC1. (A) LC-MS analysis of the reaction products obtained after 0, 90, and 180 min from the RC1 resting cells using α-tomatine as a substrate. The degradation activities measured in one replicate (n =1) are shown. A total ion current chromatogram obtained in the positive ionization mode with a full-scan range of *m/z* 300–1200 is shown. The mass spectra of peak a (substrate, α-tomatine), peaks b and c (reaction products), and peak d (tomatidine standard), as indicated by arrows in the chromatogram, are shown. The red letters represent the parental ion mass given by respective peaks. (B) Proposed metabolic conversion of α-tomatine to tomatidine, as predicted by the mass spectra of the reaction products.

FIG S3

```
                   10         20         30         40         50         60
ASV       ACGGAGGGG G CTAGCGTTG T TCGGAATTA C TGGGCGTAA A GCGCACGTA G GCGGCGATT T
RC1       ......... . ......... . ......... . ......... . ......... . ......... .
TomMM7A   ......... . ......... . ......... . ......... . ......... . ......... .
TomMM35A  ......... . ......... . ......... . ......... . ......... . ......... .
TomTYG43  ......... . ......... . ......... . ......... . ......... . ......... .
TomTYG73  ......... . ......... . ......... . ......... . ......... . ......... .
TomTYG75  ......... . ......... . ......... . ......... . ......... . ......... .
TomTYG71  ......... . ......... . ......... . ......... . ......... . ......... .
TomTYG64  ......... . ......... . ......... . ......... . ......... . ......... .
TomTYG46  ......... . ......... . ......... . ......... . ......... . ......... .
TomTYG45  ......... . ......... . ......... . ......... . ......... . ......... .
TomTYG42  ......... . ......... . ......... . ......... . ......... . ......... .
TomTYG65  ......... . ......... . ......... . ......... . ......... . ......... .
TomTYG72  ......... . ......... . ......... . ......... . ......... . ......... .
TomTYG74  ......... . ......... . ......... . ......... . ......... . ......... .
TomMM15   ......... . ......... . ......... . ......... . ......... . ......... .

                   70         80         90        100        110        120
ASV       AAGTCAGAG G TGAAAGCCC G GGGCTCAAC C CCGGAACTG C CTTTGAGAC T GGATTGCTT G
RC1       ......... . ......... . ......... . ......... . ......... . ......... .
TomMM7A   ......... . ......... . ......... . ......... . ......... . ......... .
TomMM35A  ......... . ......... . ......... . ......... . ......... . ......... .
TomTYG43  ......... . ......... . ......... . ......... . ......... . ......... .
TomTYG73  ......... . ......... . ......... . ......... . ......... . ......... .
TomTYG75  ......... . ......... . ......... . ......... . ......... . ......... .
TomTYG71  ......... . ......... . ......... . ......... . ......... . ......... .
TomTYG64  ......... . ......... . ......... . ......... . ......... . ......... .
TomTYG46  ......... . ......... . ......... . ......... . ......... . ......... .
TomTYG45  ......... . ......... . ......... . ......... . ......... . ......... .
TomTYG42  ......... . ......... . ......... . ......... . ......... . ......... .
TomTYG65  ......... . ......... . ......... . ......TA. . ......... . ......... .
TomTYG72  ......... . ......... . ......... . ......... . ......... . ......... .
TomTYG74  ......... . ......... . ......... . ......... . ......... . ......... .
TomMM15   ......... . ......... . ......... . ......... . ......... . ......... .

                  130        140        150        160        170        180
ASV       AACATCGGA G AGGTGAGTG G AATTCCGAG T GTAGAGGTG A AATTCGTAG A TATTCGGAA G
RC1       ......... . ......... . ......... . ......... . ......... . ......... .
TomMM7A   ......... . ......... . ......... . ......... . ......... . ......... .
TomMM35A  ......... . ......... . ......... . ......... . ......... . ......... .
TomTYG43  ......... . ......... . ......... . ......... . ......... . ......... .
TomTYG73  ......... . ......... . ......... . ......... . ......... . ......... .
TomTYG75  ......... . ......... . ......... . ......... . ......... . ......... .
TomTYG71  ......... . ......... . ......... . ......... . ......... . ......... .
TomTYG64  ......... . ......... . ......... . ......... . ......... . ......... .
TomTYG46  ......... . ......... . ......... . ......... . ......... . ......... .
TomTYG45  ......... . ......... . ......... . ......... . ......... . ......... .
TomTYG42  ......... . ......... . ......... . ......... . ......... . ......... .
TomTYG65  ......... . ......... . ......... . ......... . ......... . ......... .
TomTYG72  ..TC.T... . ...C.G... . ......... . ......... . ......... . ......... .
TomTYG74  ..TC.T... . ...C.G... . ......... . ......... . ......... . ......... .
TomMM15   ..TC.T... . ...C.G... . ......... . ......... . ......... . ......... .

                  190        200        210        220        230        240
ASV       AACACCAGT G GCGAAGGCG G CTCACTGGA C GATTGTTGA C GCTGAGGTG C GAAAGCGTG G
RC1       ......... . ......... . ......... . ......... . ......... . ......... .
TomMM7A   ......... . ......... . ......... . ......... . ......... . ......... .
TomMM35A  ......... . ......... . ......... . ......... . ......... . ......... .
TomTYG43  ......... . ......... . ......... . ......... . ......... . ......... .
TomTYG73  ......... . ......... . ......... . ......... . ......... . ......... .
TomTYG75  ......... . ......... . ......... . ......... . ......... . ......... .
TomTYG71  ......... . ......... . ......... . ......... . ......... . ......... .
TomTYG64  ......... . ......... . ......... . ......... . ......... . ......... .
TomTYG46  ......... . ......... . ......... . ......... . ......... . ......... .
TomTYG45  ......... . ......... . ......... . ......... . ......... . ......... .
TomTYG42  ......... . ......... . ......... . ......... . ......... . ......... .
TomTYG65  ......... . ......... . ......... . ......... . ......... . ......... .
TomTYG72  ......... . ......... . .C.G..... . A.G.A.... . ......... . ......... .
TomTYG74  ......... . ......... . .C.G..... . A.G.A.... . ......... . ......... .
TomMM15   ......... . ......... . .C.G..... . A.G.A.... . ......... . ......... .

                  250
ASV       GGAGCAAAC A GG
RC1       ......... . ..
TomMM7A   ......... . ..
TomMM35A  ......... . ..
TomTYG43  ......... . ..
TomTYG73  ......... . ..
TomTYG75  ......... . ..
TomTYG71  ......... . ..
TomTYG64  ......... . ..
TomTYG46  ......... . ..
TomTYG45  ......... . ..
TomTYG42  ......... . ..
TomTYG65  ......... . ..
TomTYG72  ......... . ..
TomTYG74  ......... . ..
TomMM15   ......... . ..
```

FIG S3. DNA sequence alignment of the 16S rRNA V4 region in *Sphingobium* isolates and the amplicon sequence variant enriched in steroid-type saponin-treated soils and tomato rhizosphere soil (28, 41).

FIG S4

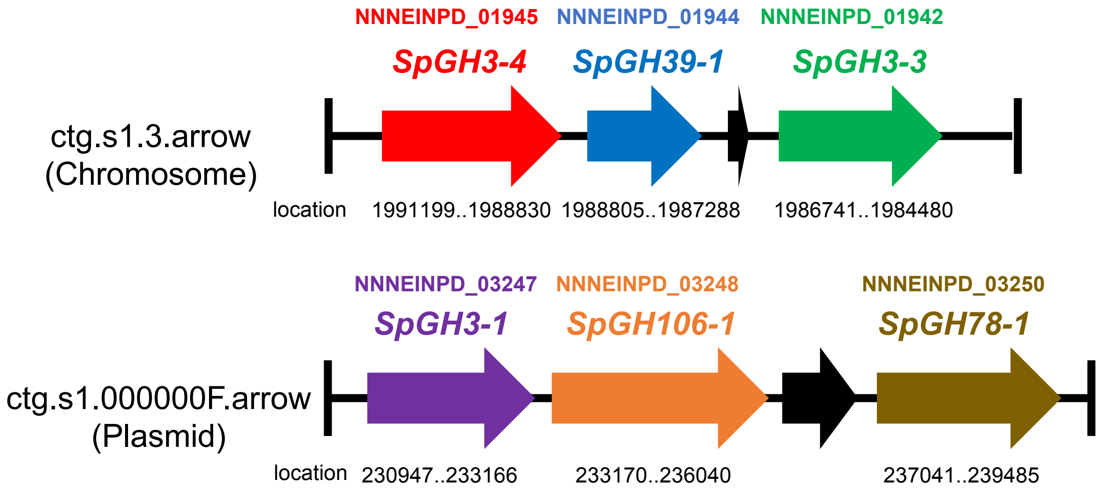

FIG S4. Organization of six glycoside hydrolase genes on two regions of the RC1 genome. The labels on the left indicate contigs of the RC1 genome. The numbers under the filled arrows represent the location of each gene.

FIG S5

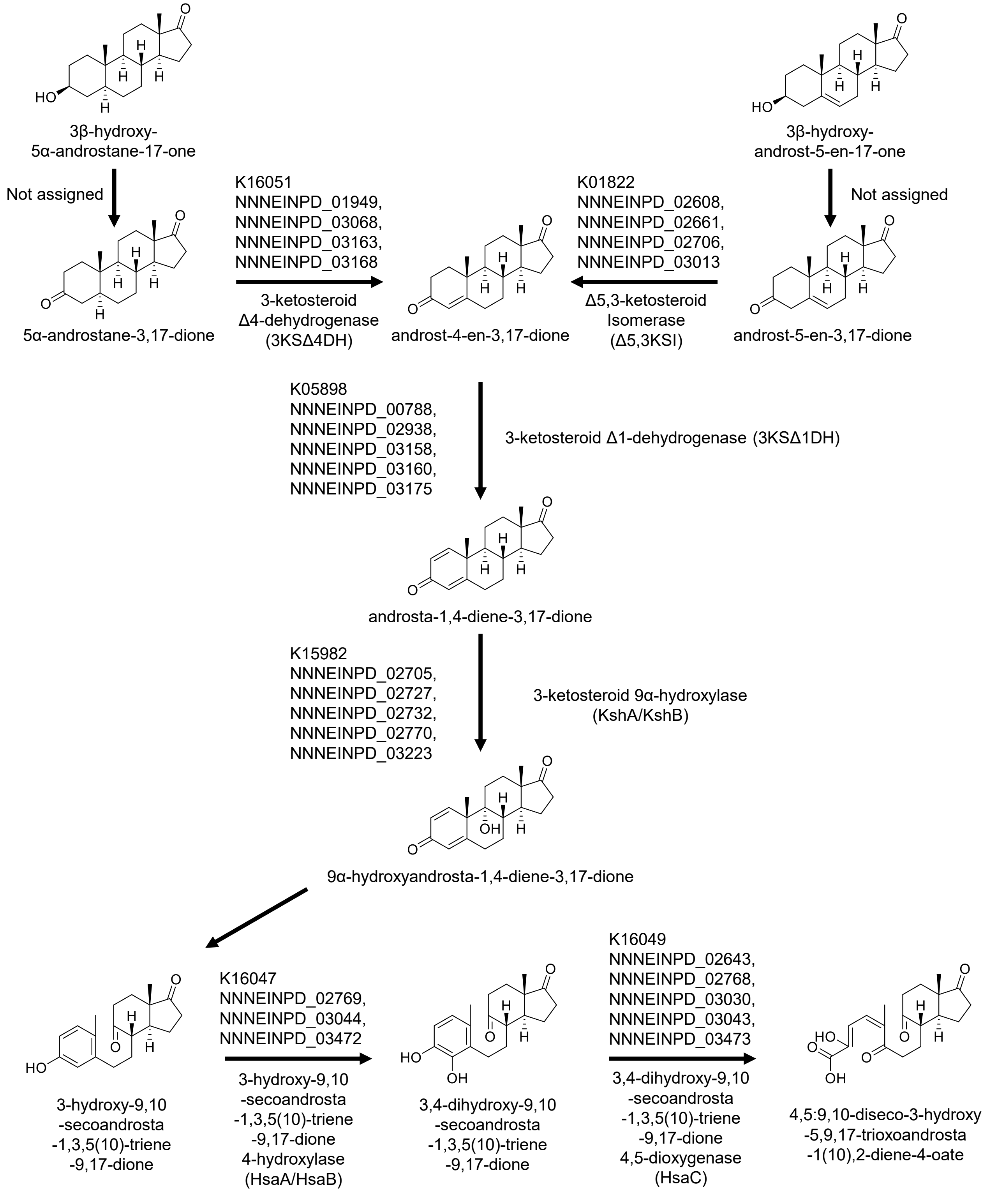

FIG S5. Functional annotation using KofamKOALA assigned several proteins in RC1 to the KEGG pathway map for steroid degradation. K numbers, KEGG Ortholog IDs. NNNEINPD numbers, locus tags of RC1 genes annotated by the Prokka pipeline.

FIG S6

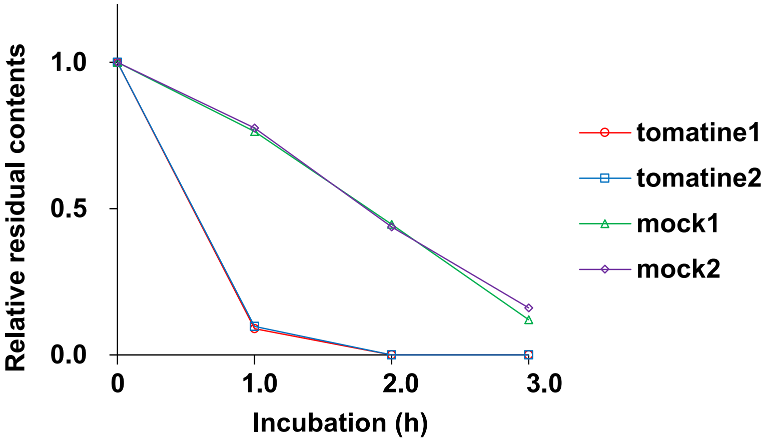

FIG S6. α-Tomatine-degradation activities in resting cells of α-tomatine- and mock-treated RC1 cells. α-Tomatine- and mock-treatments were performed in technical duplicates (tomatine1-2 and mock1-2, respectively). The residual substrate contents in the reaction mixture are shown relative to that recorded at the onset of the reaction. The degradation activities measured in one replicate (n =1) are shown.

FIG S7

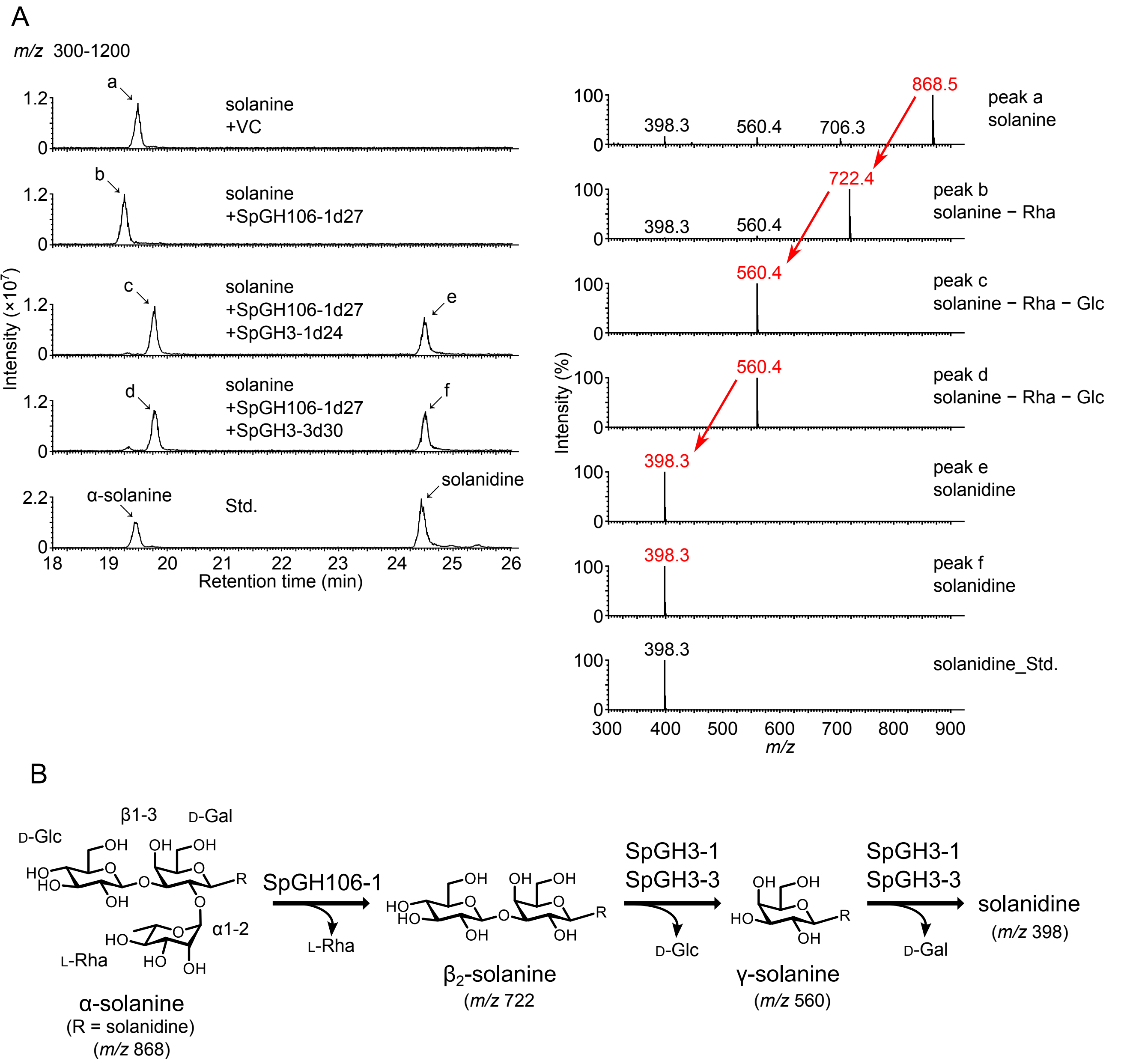

FIG S7. Enzymatic activities of SpGH3-1, SpGH3-3, and SpGH106-1 toward α-solanine. (A) LC-MS analysis of the reaction products obtained from the recombinant proteins of SpGH3-1, SpGH3-3, and SpGH106-1 using α-solanine as a substrate. A purified protein from *Escherichia coli* transformed with an empty pET22b vector was used as the negative control (VC). Representative data of the enzymatic activities measured in biological duplicates are shown. The total ion current chromatogram obtained in the positive ionization mode with a full-scan range of *m/z* 300–1200 is shown. The mass spectra of peak a (substrate, α-solanine), peaks b–f (reaction products), and solanidine standard, as indicated by arrows in the chromatogram, are shown. The red letters represent the parental ion mass of the reaction products. (B) The proposed enzymatic conversion of α-solanine to solanidine, as predicted by the mass spectra of the reaction products.

FIG S8

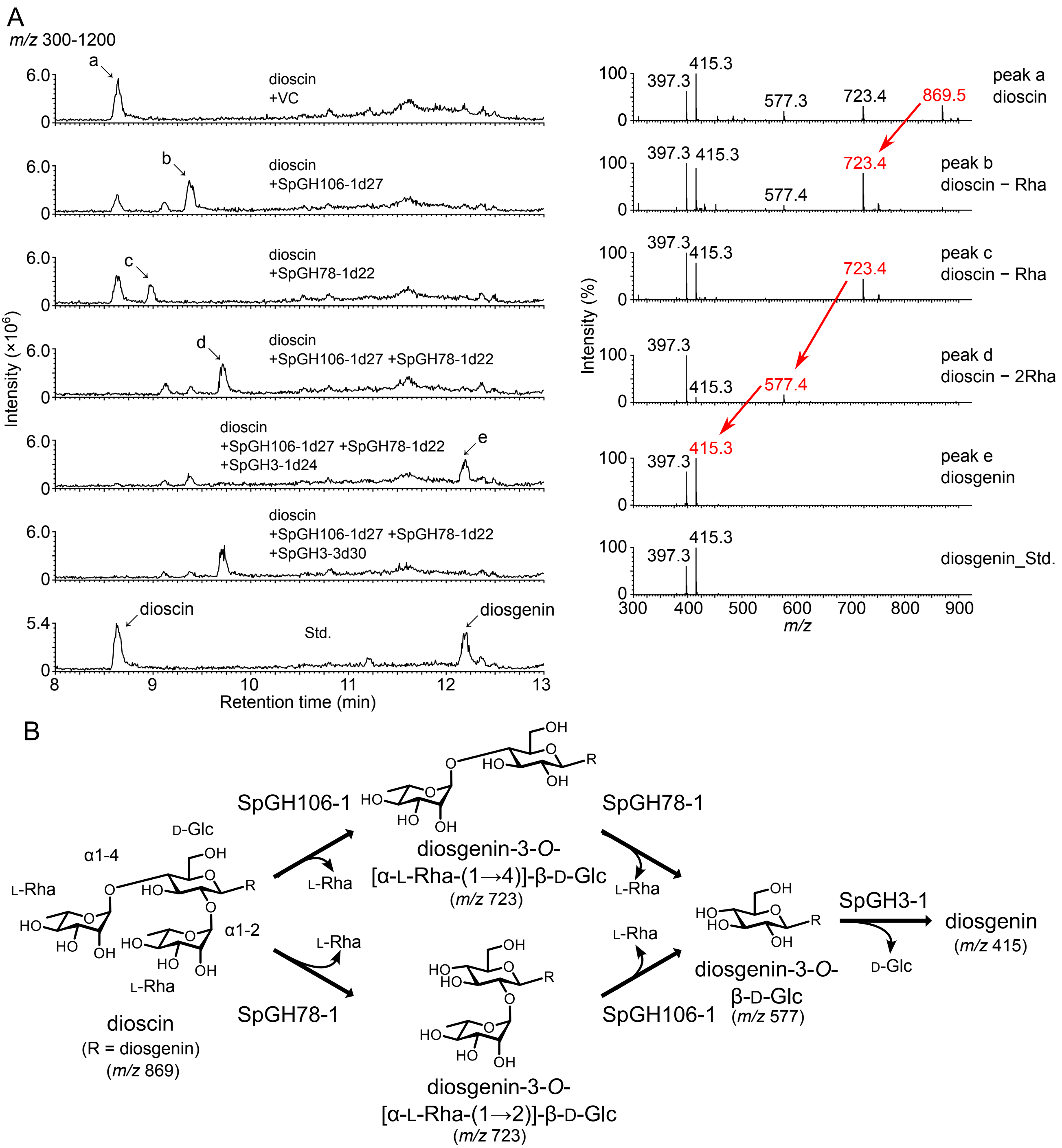

FIG S8. Enzymatic activities of SpGH3-1, SpGH3-3, SpGH78-1, and SpGH106-1 toward dioscin. (A) LC-MS analysis of the reaction products obtained from the recombinant proteins of SpGH3-1, SpGH3-3, SpGH78-1, and SpGH106-1 using dioscin as a substrate. A purified protein from *Escherichia coli* transformed with an empty pET22b vector was used as the negative control (VC). Representative data of the enzymatic activities measured in biological duplicates are shown. The total ion current chromatogram obtained in the positive ionization mode with a full-scan range of *m/z* 300–1200 is shown. The mass spectra of peak a (substrate, dioscin), peaks b–e (reaction products), and the diosgenin standard, as indicated by arrows in the chromatogram, are shown. The red letters represent the parental ion mass given by the reaction products. (B) Proposed enzymatic conversion of dioscin to diosgenin, as predicted by the mass spectra of the reaction products.

FIG S9

A

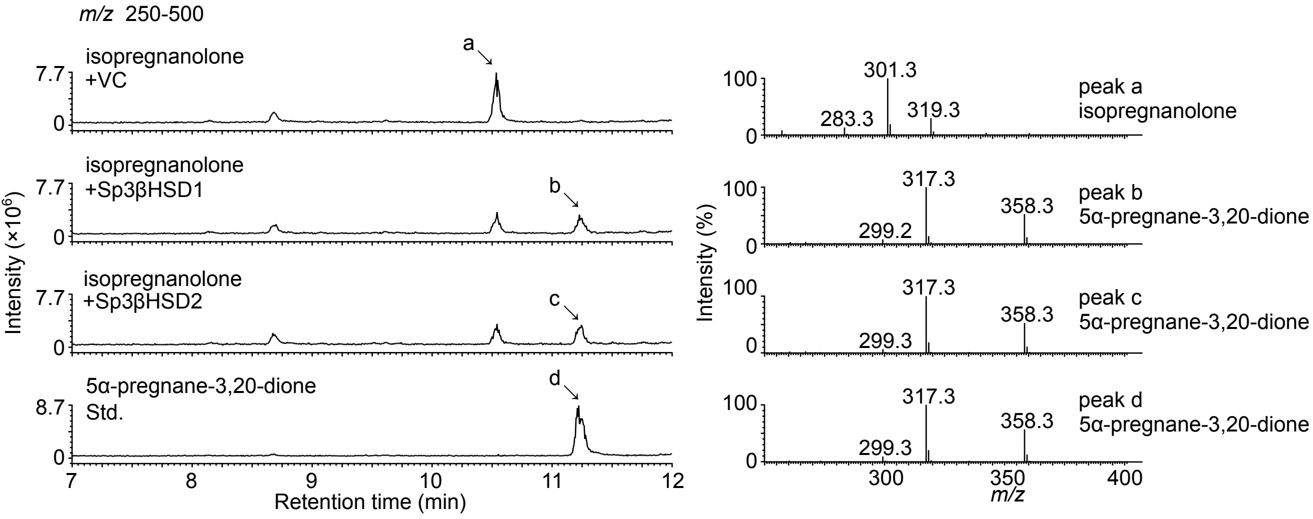

B

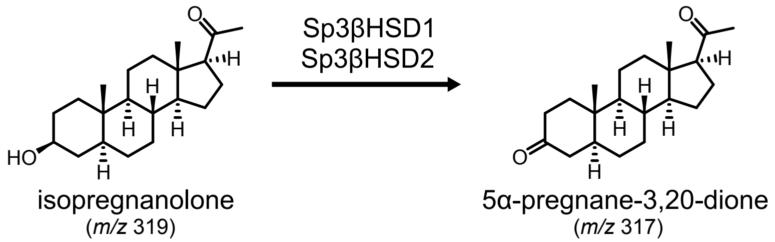

FIG S9. Enzymatic activities of Sp3βHSD1 and Sp3βHSD2 toward isopregnanolone. (A) LC-MS analysis of the reaction products obtained from the recombinant proteins of Sp3βHSD1 and Sp3βHSD2 using isopregnanolone as a substrate. A purified protein from *Escherichia coli* transformed with an empty pET22b vector was used as the negative control (VC). Representative data of the enzymatic activities measured in biological duplicates are shown. The total ion current chromatogram obtained in the positive ionization mode with a full-scan range of *m/z* 250–500 is shown. The mass spectra of peak a (substrate, isopregnanolone), peaks b and c (reaction products), and peak d (5α-pregnane-3,20-dione), as indicated by arrows in the chromatogram, are shown. (B) Enzymatic conversion of isopregnanolone to 5α-pregnane-3,20-dione.

FIG S10

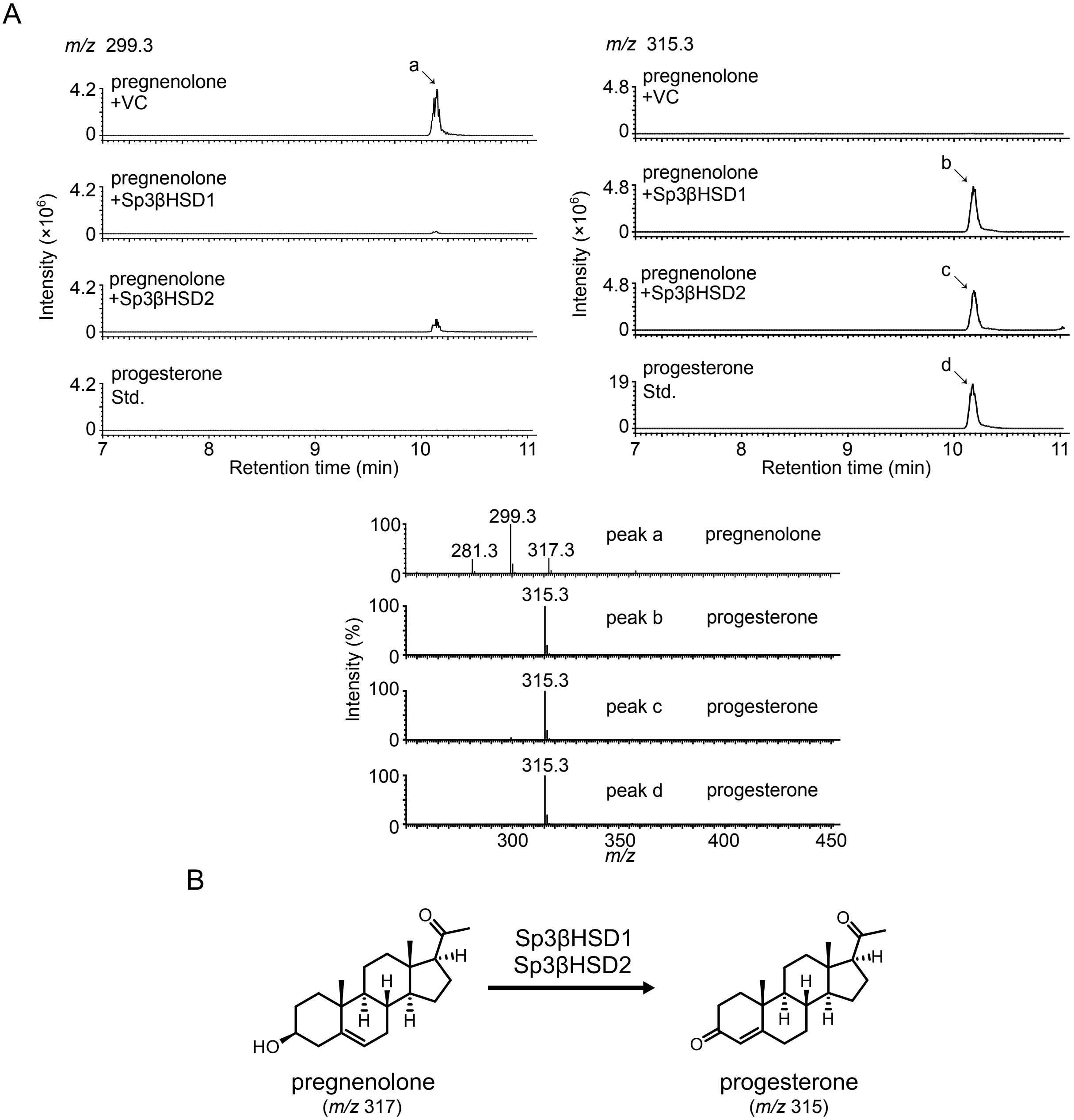

FIG S10. Enzymatic activities of Sp3βHSD1 and Sp3βHSD2 toward pregnenolone. (A) LC-MS analysis of the reaction products obtained from the recombinant proteins of Sp3βHSD1 and Sp3βHSD2 using pregnenolone as a substrate. A purified protein from *Escherichia coli* transformed with an empty pET22b vector was used as the negative control (VC). Representative data of the enzymatic activities measured in biological duplicates are shown. The extracted ion current chromatograms for *m/z* 299.3 and 315.3 obtained in the positive ionization mode with a full-scan range of *m/z* 250–500 are shown. The mass spectra of peak a (substrate, pregnenolone), peaks b and c (reaction products), and peak d (progesterone), as indicated by arrows in the chromatogram, are shown. (B) Enzymatic conversion of pregnenolone to progesterone.

FIG S11

A

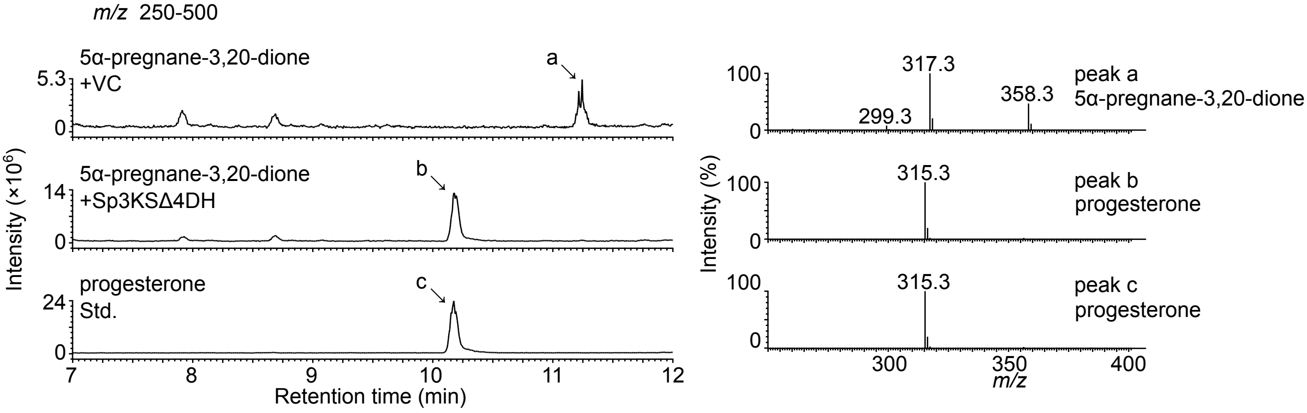

B

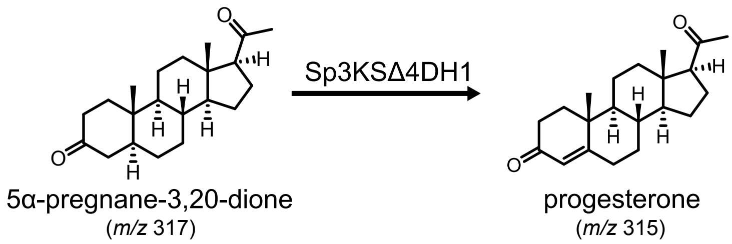

FIG S11. Enzymatic activity of Sp3KSΔ4DH1 toward 5α-pregnane-3,20-dione. (A) LC-MS analysis of the reaction products obtained from the recombinant protein of Sp3KSΔ4DH1 using 5α-pregnane-3,20-dione as a substrate. A purified protein from *Escherichia coli* transformed with an empty pET22b vector was used as the negative control (VC). Representative data of the enzymatic activities measured in biological duplicates are shown. The total ion current chromatogram obtained in the positive ionization mode with a full-scan range of *m/z* 250–500 is shown. The mass spectra of peak a (substrate, 5α-pregnane-3,20-dione), peak b (reaction product), and peak c (progesterone), as indicated by arrows in the chromatogram, are shown. (B) Enzymatic conversion of 5α-pregnane-3,20-dione to progesterone.

FIG S12

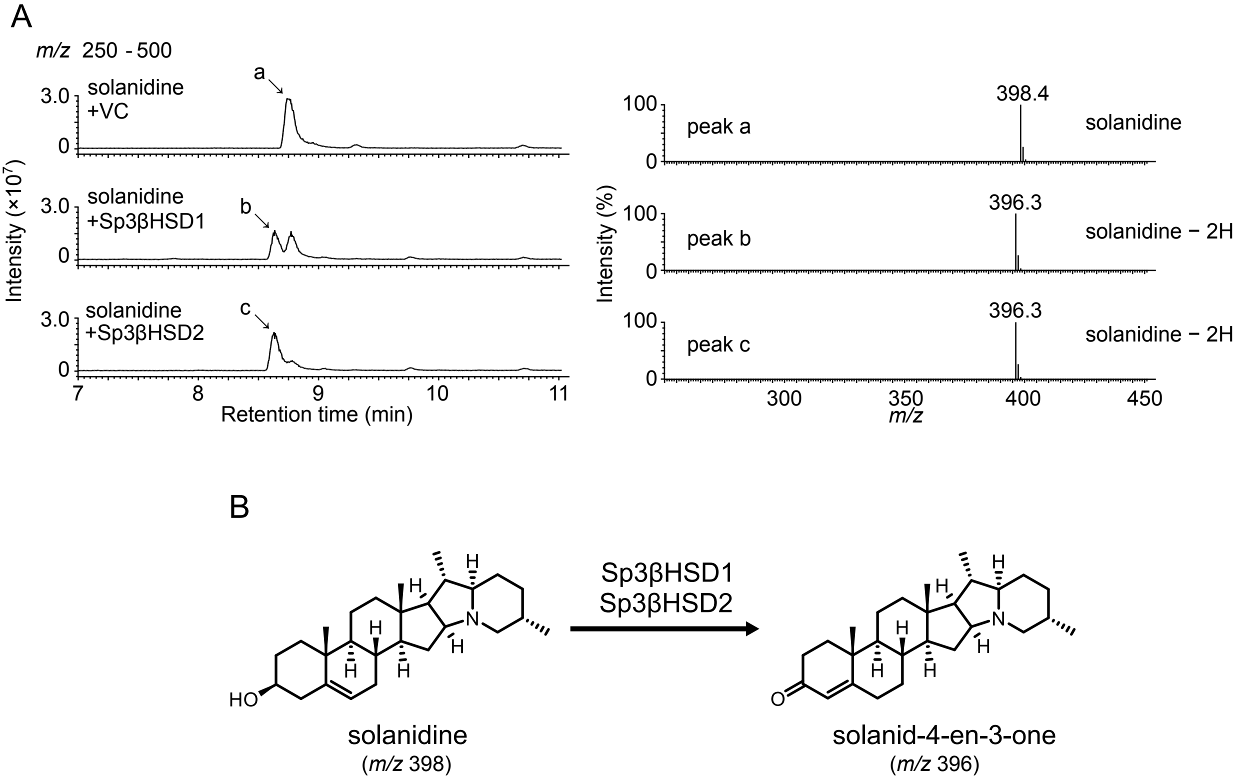

FIG S12. Enzymatic activities of Sp3βHSD1 and Sp3βHSD2 toward solanidine. (A) LC-MS analysis of the reaction products obtained from the recombinant proteins of Sp3βHSD1 and Sp3βHSD2 using solanidine as a substrate. A purified protein from *Escherichia coli* transformed with an empty pET22b vector was used as the negative control (VC). Representative data of the enzymatic activities measured in biological duplicates are shown. The total ion current chromatogram obtained in the positive ionization mode with a full-scan range of *m/z* 250–500 is shown. The mass spectra of peak a (substrate, solanidine) and peaks b and c (reaction products), as indicated by arrows in the chromatogram, are shown. (B) Proposed enzymatic conversion of solanidine to solanid-4-en-3-one, as predicted by the enzymatic activities of Sp3βHSD1 and Sp3βHSD2 toward pregnenolone (Fig. S10).

FIG S13

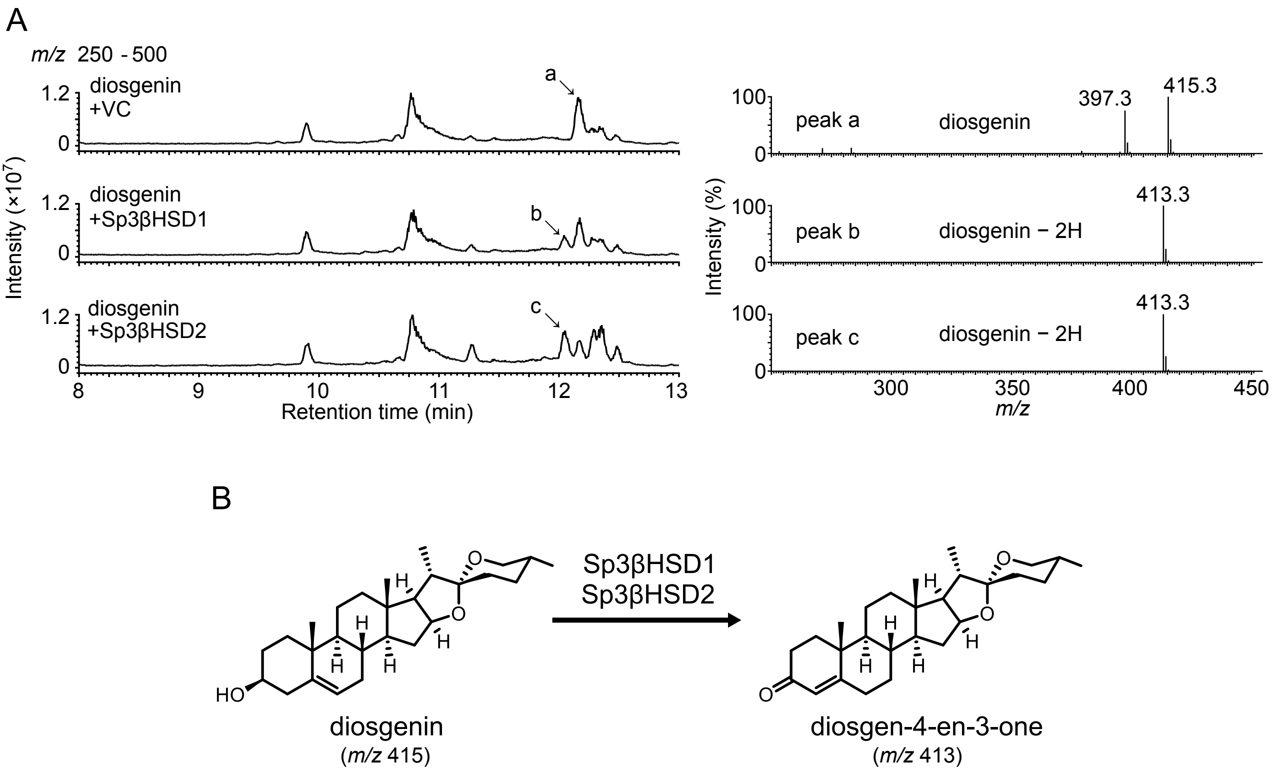

FIG S13. Enzymatic activities of Sp3βHSD1 and Sp3βHSD2 toward diosgenin. (A) LC-MS analysis of the reaction products obtained from the recombinant proteins of Sp3βHSD1 and Sp3βHSD2 using diosgenin as a substrate. A purified protein from *Escherichia coli* transformed with an empty pET22b vector was used as the negative control (VC). Representative data of the enzymatic activities measured in biological duplicates are shown. The total ion current chromatogram obtained in the positive ionization mode with a full-scan range of *m/z* 250–500 is shown. The mass spectra of peak a (substrate, diosgenin) and peaks b and c (reaction products), as indicated by arrows in the chromatogram, are shown. (B) Proposed enzymatic conversion of diosgenin to diosgen-4-en-3-one, as predicted by the enzymatic activities of Sp3βHSD1 and Sp3βHSD2 toward pregnenolone (Fig. S10).

FIG S14

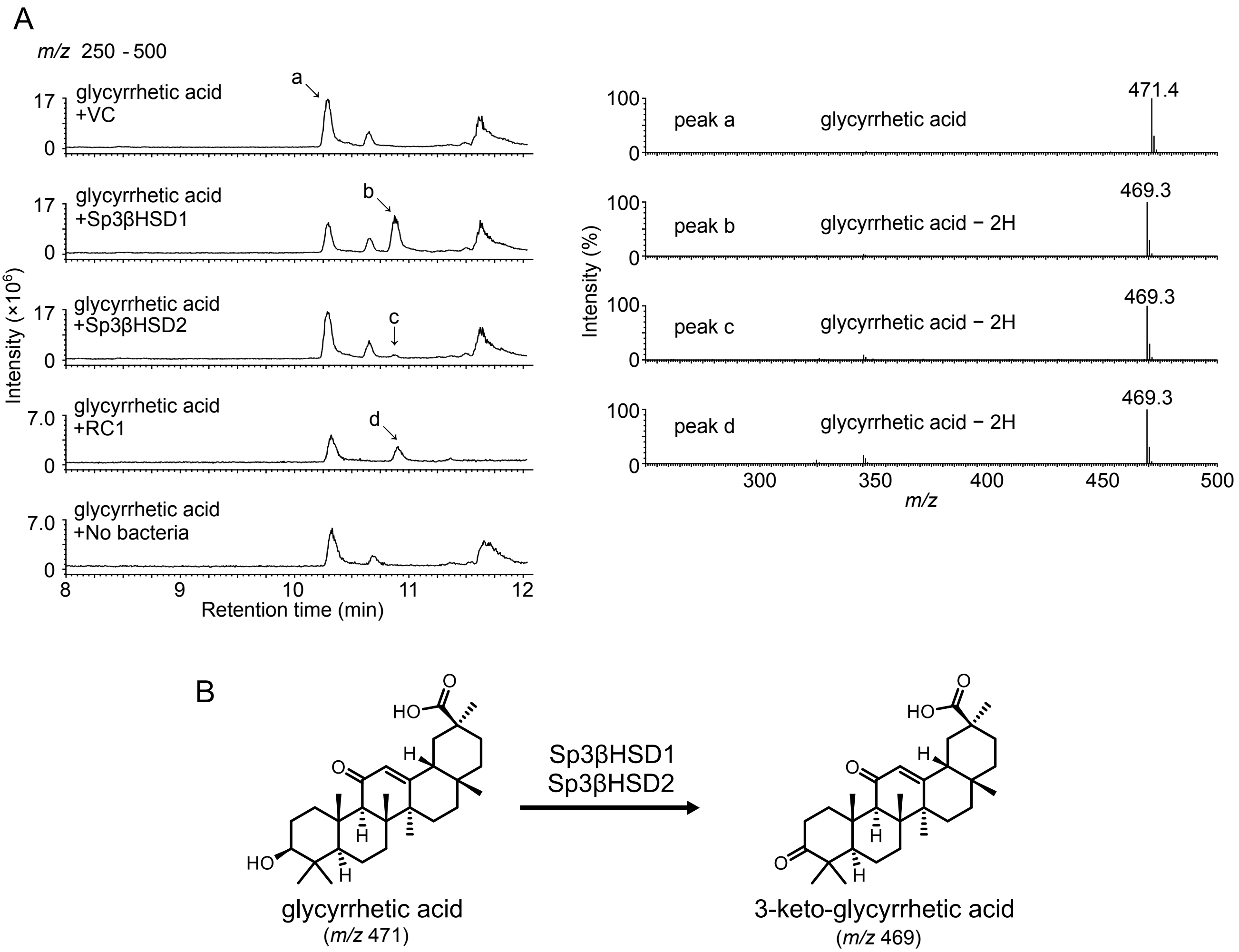

FIG S14. Enzymatic activities of Sp3βHSD1 and Sp3βHSD2 toward glycyrrhetic acid. (A) LC-MS analysis of the reaction products obtained from the recombinant proteins of Sp3βHSD1 and Sp3βHSD2 and RC1 resting cells using glycyrrhetic acid as a substrate. A purified protein from *Escherichia coli* transformed with an empty pET22b vector was used as the negative control (VC). Representative data of the enzymatic activities measured in biological duplicates are shown. The total ion current chromatogram obtained in the positive ionization mode with a full-scan range of *m/z* 250–500 is shown. The mass spectra of peak a (substrate, glycyrrhetic acid) and peaks b–d (reaction products), as indicated by arrows in the chromatogram, are shown. (B) Proposed enzymatic conversion of glycyrrhetic acid to 3-keto-glycyrrhetic acid, as predicted by the mass spectra of the reaction products.

FIG S15

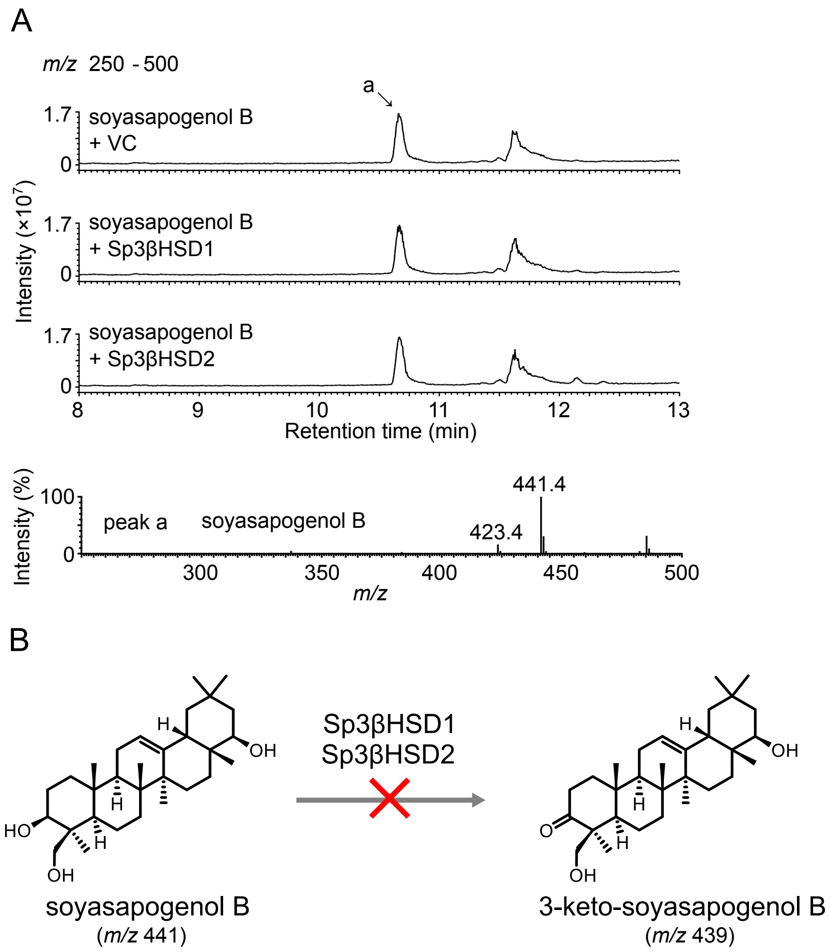

FIG S15. Enzymatic activities of Sp3βHSD1 and Sp3βHSD2 toward soyasapogenol B. (A) LC-MS analysis of the reaction products obtained from the recombinant proteins of Sp3βHSD1 and Sp3βHSD2 and RC1 resting cells using soyasapogenol B as a substrate. A purified protein from *Escherichia coli* transformed with an empty pET22b vector was used as the negative control (VC). Representative data of the enzymatic activities measured in biological duplicates are shown. The total ion current chromatogram obtained in the positive ionization mode with a full-scan range of *m/z* 250–500 is shown. The mass spectrum of peak a (substrate, soyasapogenol B), as indicated by the arrow in the chromatogram, is shown. (B) Neither Sp3βHSD1 nor Sp3βHSD2 metabolized soyasapogenol B to 3-keto- soyasapogenol B.

FIG S16

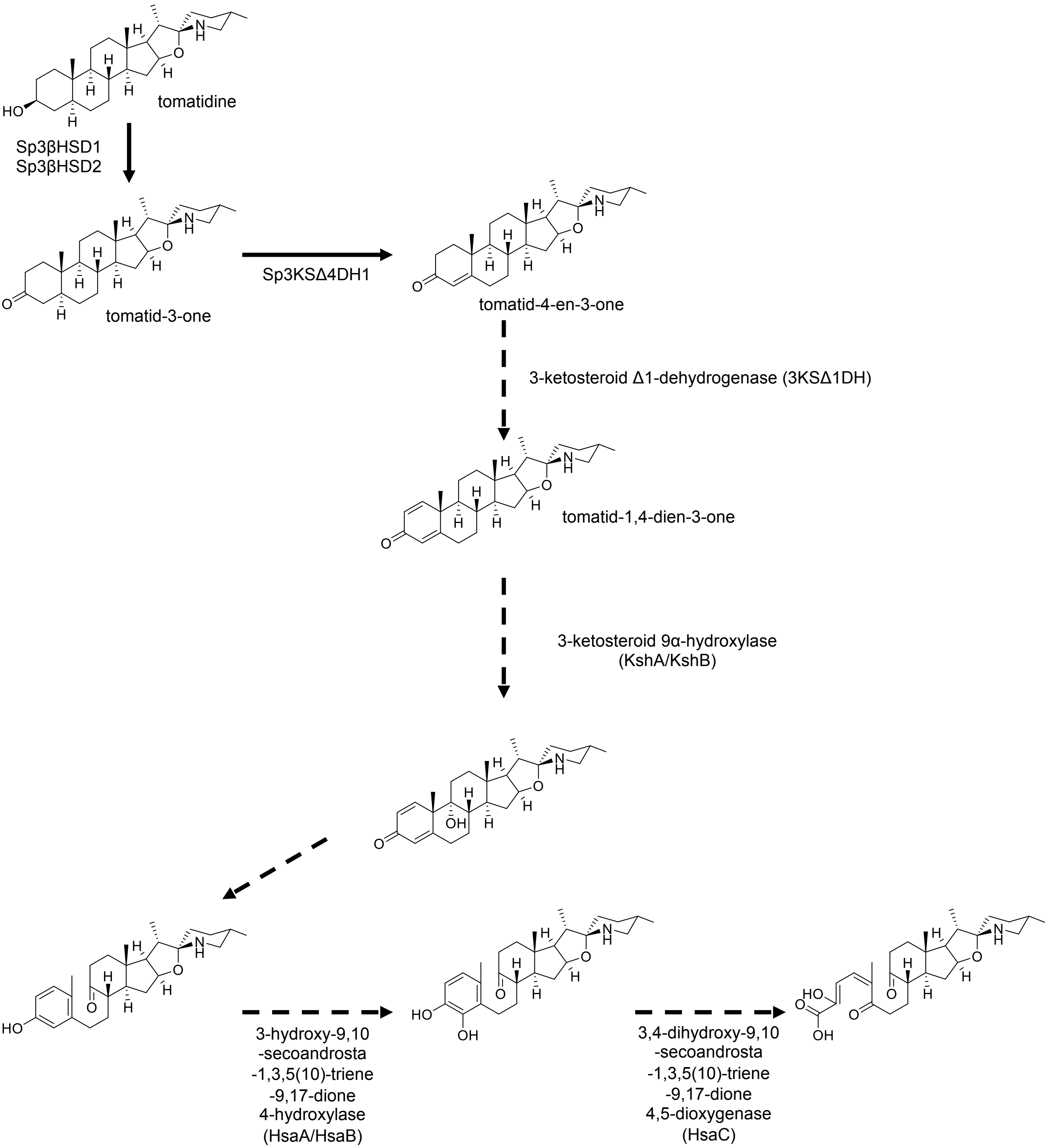

FIG S16. The putative tomatidine degradation pathway proposed in this study. Solid arrows indicate the reaction steps identified in this work. Dashed arrows indicate reaction stages suggested based on the known steroid degradation pathway.

TABLE S1 *Sphingobium* isolates used in this study.

Isolate names	medium (agar plate)	source
RC1	tryptone yeast extract glucose	tomato roots
TomMM7A	mineral salt buffer containing 20 mg/mL of tomatidine	α-tomatine-treated soil
TomMM35A	mineral salt buffer containing 20 mg/mL of tomatidine	α-tomatine-treated soil
TomMM15	mineral salt buffer containing 20 mg/mL of α-tomatine	α-tomatine-treated soil
TomTYG42	tryptone yeast extract glucose	α-tomatine-treated soil
TomTYG43	tryptone yeast extract glucose	α-tomatine-treated soil
TomTYG45	tryptone yeast extract glucose	α-tomatine-treated soil
TomTYG46	tryptone yeast extract glucose	α-tomatine-treated soil
TomTYG64	tryptone yeast extract glucose	α-tomatine-treated soil
TomTYG65	tryptone yeast extract glucose	α-tomatine-treated soil
TomTYG71	tryptone yeast extract glucose	α-tomatine-treated soil
TomTYG72	tryptone yeast extract glucose	α-tomatine-treated soil
TomTYG73	tryptone yeast extract glucose	α-tomatine-treated soil
TomTYG74	tryptone yeast extract glucose	α-tomatine-treated soil
TomTYG75	tryptone yeast extract glucose	α-tomatine-treated soil

TABLE S2 Transcript levels (transcripts per million, TPM) of saponin-degrading candidates in α-tomatine- and mock-treated RC1. The TPM values of all genes plus 0.01 was used to calculate the ratio of the transcript levels.

Gene IDs	mock1	mock2	tomatine1	tomatine2	ratio of tomatine to mock	Gene names	KEGG orthology in the steroid degradation pathway
NNNEINPD_01942	0.8	1.1	31.8	24.2	29.2	SpGH3-3	
NNNEINPD_01944	0.7	0.6	50.7	33.2	64.9	SpGH39-1	
NNNEINPD_01945	0.7	0.9	27.0	14.1	25.4	SpGH3-4	
NNNEINPD_03247	0.5	1.3	6.8	5.4	6.6	SpGH3-1	
NNNEINPD_03248	1.0	1.8	14.8	11.3	9.0	SpGH106-1	
NNNEINPD_03250	1.3	1.9	18.0	14.0	10.0	SpGH78-1	
NNNEINPD_02694	0.9	2.7	21.3	17.4	10.8	Sp3βHSD2	
NNNEINPD_03057	0.0	0.4	3.8	3.8	19.9	Sp3βHSD1	
NNNEINPD_02706	5.3	1.1	22.9	7.0	4.7		K01822 (Δ5,3-KSI)
NNNEINPD_02661	0.7	1.0	25.0	14.6	22.9		K01822 (Δ5,3-KSI)
NNNEINPD_03013	0.7	1.0	25.0	14.6	22.9		K01822 (Δ5,3-KSI)
NNNEINPD_02608	0.7	1.0	25.0	14.6	22.9		K01822 (Δ5,3-KSI)
NNNEINPD_01949	1.4	1.6	6.7	6.7	4.5	Sp3KSΔ4DH1	K16051 (3KSΔ4DH)
NNNEINPD_03163	2.8	2.0	7.3	6.8	3.0		K16051 (3KSΔ4DH)
NNNEINPD_03068	1.3	1.0	9.9	9.6	8.4		K16051 (3KSΔ4DH)
NNNEINPD_03168	0.2	1.4	1.9	2.0	2.5		K16051 (3KSΔ4DH)
NNNEINPD_02938	1.1	0.9	36.2	20.4	28.6		K05898 (3KSΔ1DH)
NNNEINPD_00788	0.8	0.9	5.6	1.2	3.9		K05898 (3KSΔ1DH)
NNNEINPD_03158	0.2	0.3	3.0	3.9	13.0		K05898 (3KSΔ1DH)
NNNEINPD_03160	0.2	0.4	1.5	2.7	6.4		K05898 (3KSΔ1DH)
NNNEINPD_03175	1.3	1.1	4.4	3.1	3.1		K05898 (3KSΔ1DH)
NNNEINPD_02770	0.8	0.3	0.9	3.1	3.7		K15982 (KshA)
NNNEINPD_03223	2.3	3.1	3.9	2.3	1.1		K15982 (KshA)
NNNEINPD_02705	0.8	5.3	36.7	27.3	10.4		K15982 (KshA)
NNNEINPD_02732	28.7	27.0	148.4	103.1	4.5		K15982 (KshA)
NNNEINPD_02727	6.0	7.1	9.6	6.4	1.2		K15982 (KshA)
NNNEINPD_03044	3.3	4.0	78.6	58.1	18.7		K16047 (HsaA)
NNNEINPD_02769	0.9	0.2	0.9	1.3	2.1		K16047 (HsaA)
NNNEINPD_03472	3.3	4.0	78.6	58.1	18.7		K16047 (HsaA)
NNNEINPD_03043	0.8	1.2	14.3	12.1	13.2		K16049 (HsaC)
NNNEINPD_02768	1.4	0.6	2.5	2.1	2.3		K16049 (HsaC)
NNNEINPD_03473	0.8	1.2	14.3	12.1	13.2		K16049 (HsaC)
NNNEINPD_03030	0.8	1.2	14.3	12.1	13.2		K16049 (HsaC)
NNNEINPD_02643	0.8	1.2	14.3	12.1	13.2		K16049 (HsaC)

TABLE S3 Substrate specificity of six saponin glycoside hydrolases. Representative data of the enzymatic activities measured in biological duplicates are shown.

	α-tomatine	α-solanine	dioscin	soyasaponin Bb	glycyrrhizin
SpGH3-1	N.D.	N.D.	N.D.	N.D.	N.D.
SpGH3-3	N.D.	N.D.	N.D.	N.D.	N.D.
SpGH3-4	+	N.D.	N.D.	N.D.	N.D.
SpGH39-1	+	N.D.	N.D.	N.D.	N.D.
SpGH78-1	N.D.	N.D.	+	N.D.	N.D.
SpGH106-1	N.D.	+	+	N.D.	N.D.
